# Cross-sectional and longitudinal associations of active travel, organised sport and physical education with accelerometer-assessed moderate-to-vigorous physical activity in young people: the International Children’s Accelerometry Database

**DOI:** 10.1186/s12966-022-01282-4

**Published:** 2022-04-02

**Authors:** Erika Ikeda, Justin M. Guagliano, Andrew J. Atkin, Lauren B. Sherar, Ulf Ekelund, Bjørge Hansen, Kate Northstone, Esther van Sluijs, On behalf of the International Children’s Accelerometry Database (ICAD) Collaborators, Jo Salmon, Chris Riddoch, Ken Judge, Ashley Cooper, Pippa Griew, L. B. Andersen, S. Anderssen, G. Cardon, R. Davey, P. Hallal, R. Jago, K. F. Janz, S. Kriemler, N. Møller, K. Northstone, R. Pate, J. J. Puder, J. Reilly, J. Salmon, L. B. Sardinha, E. M. F. van Sluijs

**Affiliations:** 1 MRC Epidemiology Unit, University of Cambridge, Cambridge, UK; 2 School of Health Sciences, Western Sydney University, Sydney, Australia; 3 School of Health Sciences, University of East Anglia, Norwich, UK; 4 Norwich Epidemiology Centre, University of East Anglia, Norwich, UK; 5 School of Sport, Exercise, and Health Sciences, Loughborough University, Loughborough, UK; 6 Department of Sports Medicine, Norwegian School of Sport Sciences, Oslo, Norway; 7 Department of Sport Science and Physical Education, University of Agder, Kristiansand, Norway; 8 Population Health Sciences, Bristol School of Medicine, University of Bristol, Bristol, UK

**Keywords:** Active transport, Adolescent, Children, ICAD, MVPA, Organized sport, PE

## Abstract

**Background:**

Physical activity (PA) declines during childhood. Important sources of PA are active travel, organised sport and physical education (PE), but it is unclear how these domain-specific PA sources contribute to (changes in) daily moderate-to-vigorous PA (MVPA) in young people. This study aimed to examine (1) the cross-sectional association between domain-specific physical activity (i.e., active travel, organised sport and PE) and daily minutes in accelerometer-assessed MVPA; and (2) the longitudinal association between domain-specific physical activity at baseline and change in daily minutes in MVPA.

**Methods:**

Participants (baseline age 11.3 ± .1.2 years) were drawn from three studies in the International Children’s Accelerometry Database. The contribution of self-reported standardised active travel, organised sport and PE to accelerometer-measured daily minutes in MVPA was examined using linear regression. In cross-sectional analyses, MVPA was regressed on each PA domain in separate models, adjusted for study, age, sex, maternal education, season, and monitor wear time. In longitudinal analyses, change in MVPA was regressed on each of the baseline PA domains, additionally adjusting for changes in season and wear time, follow-up duration, and baseline MVPA. R-squared was used to compare variance explained by each PA domain.

**Results:**

In the cross-sectional analyses (*n* = 3871), organised sport (standardised β = 3.81, 95% confidence interval [95%CI] = 3.06, 4.56) and active travel (β = 3.46, 95%CI = 2.73, 4.19) contributed more to daily MVPA than PE (β = 0.82, 95%CI = -0.02, 1.66). Compared to the base model which included only covariates (R^2^ = 21.5%), organised sport (absolute change: + 1.9%) and active travel (+ 1.7%) models explained more of the variance than the PE model (± < 0.1%). Associations followed a similar pattern in the longitudinal analyses (*n* = 2302), but none of the PA domains predicted change in MVPA (organised sport: standardised β = 0.85, 95%CI = -0.03, 1.72; active travel: β = 0.68, 95%CI = -0.14, 1.50; PE: β = 0.02, 95%CI = -0.87, 0.91).

**Conclusions:**

A multi-sectoral approach covering a wide range of PA domains should be promoted to minimise the age-related decline in MVPA during childhood.

**Supplementary Information:**

The online version contains supplementary material available at 10.1186/s12966-022-01282-4.

## Background

The recent physical activity guidelines published by the World Health Organization (WHO) recommend that children and adolescents aged 5–17 years should do at least an average of 60 min of daily moderate-to-vigorous-intensity physical activity (MVPA) across the week [1, 2]. This recommendation is supported by evidence demonstrating the association between physical activity and health benefits such as physical fitness, cardiometabolic health, cognitive outcomes, mental health and body composition [3]. In general, physical activity accumulated at higher intensities (e.g., MVPA) is more strongly associated with cardiometabolic health than sedentary behaviour and light physical activity [4, 5]. Despite this evidence, the global prevalence of self-reported physical inactivity (i.e., not meeting the physical activity recommendation) among adolescents (aged 11–17 years) from 146 countries was estimated at 81% in 2016 [6]. Likewise, results from the Global Matrix 3.0 of Report Cards, which graded the best available national population-level physical activity data between 2013 and 2018 in children and adolescents (aged 5–17 years) from 49 countries, showed that more than two thirds were physically inactive [7]. These global figures demonstrate the need for prompt political and practical actions to increase physical activity among children and adolescents globally.

Among school-aged children and adolescents, physical activity occurs predominantly in the contexts of active travel (e.g., walking and cycling to school), organised sport (e.g., swimming or playing football at a club), leisure activities (e.g., play), and education (e.g., physical education (PE) at school) [8, 9]. Existing cross-sectional studies have separately explored associations between a single domain of physical activity (e.g., active travel, organised sport or PE) measured using surveys and MVPA measured using accelerometers in children and adolescents (e.g., [10–12]). They found that participation in active travel and the frequency and duration of organised sports and PE were positively associated with time spent in MVPA. However, little is known about the relative contributions of different domains of activity to MVPA cross-sectionally as well as to change in MVPA longitudinally. Understanding which type or domain of physical activity should be promoted to most efficiently increase MVPA is informative for intervention design, policy development and infrastructure investment [8].

The current study aimed to examine how active travel, organised sport and PE contribute to MVPA in school-aged children and adolescents. Specifically, we examined (1) the cross-sectional association between domain-specific physical activity (i.e., active travel, organised sport and PE) and accelerometer-assessed daily minutes in MVPA; and (2) the longitudinal association between domain-specific physical activity at baseline and change in daily minutes in MVPA. Secondary analyses explored associations for moderate (MPA) and vigorous (VPA) physical activity separately. We considered active travel, organised sport and PE as the key domains where policy actions might have the most influence [13, 14].

## Methods

The reporting of this study follows the STrengthening the Reporting of OBservational studies in Epidemiology (STROBE) guidelines [15] (Additional file 1).

### Study selection and participants

The International Children’s Accelerometry Database (ICAD) project is a consortium including 23 international studies (www.mrc-epid.cam.ac.uk/research/studies/icad/). The ICAD project pooled, processed and harmonised raw accelerometer (Actigraph) and health-related (behavioural, sociodemographic, anthropometric and metabolic) data collected on children and adolescents [16, 17]. This analysis uses data from ICAD 2.0. Three longitudinal studies included data on all three physical activity domains of interest: active travel (i.e., mode of travel to school), organised sport (i.e., weekly frequency of organised sports), and PE (i.e., weekly duration of PE). Thus, participants from the Avon Longitudinal Study of Parents and Children (ALSPAC; England) [18, 19], the Children Living in Active Neighbourhoods (CLAN; Australia) [20] and the Sport, Physical activity and Eating behaviour: Environmental Determinants in Young people (SPEEDY; England) [21] were included in the analyses. All studies conducted three waves of data collection. CLAN Wave 1 data were not included as some participants were 5–6 years of age and we assumed that the nature of activities and developmental skills (e.g., limited fundamental motor skills including running, throwing and kicking) among this population might differ from middle to late childhood and adolescence [22], which was the age-range covered by other studies included in the analysis.

### Measures

#### Physical activity

The validity of the Actigraph for measuring physical activity in children under free-living conditions has been established against the doubly labelled water method as the criterion measure [23]. Due to a mix of the older and newer generations of Actigraph accelerometers used across the studies as well as between their waves, shorter epochs were reintegrated to 60 s [17]. A period of non-wear was defined as at least 60 min of consecutive zeros, allowing for 2 minutes of non-zero interruptions. All data were analysed using KineSoft version 3.3.20 (KineSoft, Loughborough, UK). For the current analyses, a valid day was defined as at least 8 hours of wear time between 06:00 and 23:59. Participants were included if they provided ≥4 valid days (including at least 1 weekend day). Established cut points were used to classify sedentary time (0–99 counts per minute [cpm]) and physical activity intensity (light: 100–2295 cpm, moderate: 2296–4011 cpm, vigorous: ≥ 4012 cpm, and moderate-to-vigorous: ≥ 2296 cpm) [24]. Primary outcomes were (1) the average minutes of MVPA per valid day at baseline for cross-sectional analyses, and (2) change in the average minutes of MVPA per valid day from baseline to follow-up. Average daily minutes of MPA and VPA were secondary outcomes. A binary variable of meeting physical activity guidelines was also created for descriptive purposes based on the primary outcome of the average minutes of daily MVPA at baseline: *Meeting guidelines* (≥ 60 min of average daily MVPA) and *Not meeting guidelines* (< 60 min of average daily MVPA).

#### Active travel

Travel mode to school was child- or parent-reported. The current analyses used a harmonised mode of travel to school variable in which responses were categorised into *Active* mode (i.e., walk or cycle) or *Other* mode (i.e., public transport, car or other). A description of the original questionnaire items used to collect this data and the harmonisation process are included in Additional file 2.

#### Organised sport

Participation in organised sports was child- or parent-reported. For the purposes of harmonisation, organised sports were defined as being (1) formed by rules, facilities, equipment, normative beliefs and policies, and (2) formally arranged by a club, association, school (except for PE) or other types of organisation [25]. Other leisure-time physical activities such as play, bike riding and aerobics were not considered as organised sport, and therefore not included. The current analyses used the weekly frequency of organised sports (range: 0–59.5 times/week). The distribution of this variable varied by study as different numbers of organised sports were included in each study (i.e., ALSPAC: 2 activities; CLAN: 20; SPEEDY: 14). Thus, study-specific cut-offs were applied to classify this variable into five groups: *Never*, *Occasionally*, *Sometimes*, *Often* and *Usually*. Additional file 3 presents details of original questionnaire items and the harmonisation and categorisation process.

#### Physical education

Teacher-, child- or parent-reported data was used to indicate the weekly duration of PE. The responses were categorised into five groups: *0–59*, *60–89*, *90–119*, *120–149*, and *≥ 150* min/week. These cut-offs were chosen based on the distribution of the harmonised data as well as national and state governments’ recommendations of time spent on PE per week in England and Australia (Victoria) (e.g., [26–28]). Additional file 4 provides further information on harmonisation and classification of PE duration.

#### Covariates

Participant age, sex and maternal education reported at baseline were treated as continuous, binary (i.e., male, female) and nominal (i.e., high school, college, university) variables, respectively. Maternal education was used as a proxy for socio-economic position, and its harmonisation notes are available on the ICAD website (www.mrc-epid.cam.ac.uk/research/studies/icad/data-harmonisation/). Harmonised categories for maternal education were ‘compulsory education’, ‘some post-compulsory or vocational training’, and ‘undergraduate or postgraduate education’, which were labelled as *High school*, *College* and *University*, respectively.

Season was established using the month when accelerometers were deployed. Each season was determined by the Earth’s exposure to the sun (daylight) rather than meteorological seasons (weather). For the ALSPAC and SPEEDY studies (northern hemisphere), winter is in January–March, spring in April–June, summer in July–September, and autumn in October–December. For the CLAN study, the opposite seasons were applied as the study was conducted in the southern hemisphere. The four seasons (i.e., *Winter*, *Spring*, *Summer* and *Autumn*) during the baseline year were used for cross-sectional analyses. For longitudinal analyses, a new variable was generated for the difference in seasons between baseline and follow-ups. Responses were categorised into *Same*, *Shorter/colder* (i.e., spring/summer at baseline and winter/autumn at follow-up) and *Longer/warmer* (i.e., winter/autumn at baseline and spring/summer at follow-up).

Accelerometer wear time was assessed using the average minutes of valid wear time per valid day at baseline for cross-sectional analyses and change in the average minutes of valid wear time per valid day from baseline to follow-up for longitudinal analyses. In longitudinal analyses, follow-up duration was calculated in years based on elapsed time between the first day of accelerometer wear at baseline and follow-up assessments.

#### Data analyses

All statistical analyses were conducted using Stata version 16.1 (StataCorp, USA). Frequencies, percentages and summary statistics including central tendency (means, median) and dispersion (standard deviation [SD], quartiles) were calculated for categorical and continuous variables. The distributions of physical activity outcome variables were examined before inferential statistics. No imputation was performed to replace missing values as the missing time per day in accelerometer data, for example, varies across days and between participants [29]. Differences in baseline data (i.e., age, sex, maternal education, MVPA) of participants included and excluded from cross-sectional and longitudinal analyses were tested using either independent t-tests or chi-squared tests.

In both cross-sectional and longitudinal analyses, linear regression was used to obtain measures of association (i.e., regression coefficients) and R-squared (i.e., the proportion of variance in the outcome variable that is explained by the exposure variable and covariates in the model). Separate models were computed for each exposure variable (active travel, organised sport and PE), adjusting for the same covariates (age, sex, maternal education, season and monitor wear time) and study (ALSPAC, CLAN and SPEEDY). For longitudinal analyses, data from two follow-ups were modelled as repeated observations on individuals, and non-independence of these observations was accounted for using robust standard errors. In addition to the covariates listed above, longitudinal models were adjusted for follow-up duration and the outcome variable at baseline to account for the initial level of physical activity which was negatively correlated with the magnitude of change in physical activity at follow-up (e.g., MVPA: *r* = − 0.51, *p* < 0.001; MPA and VPA: *r* = − 0.50, *p* < 0.001). The exposure variables were standardised to enable direct comparison of associations between models. Standardised coefficients represent change in the outcome variable associated with a one SD change in the exposure variable. The exposure variables were not included in the same model because there may be collinearity (despite a variance inflation factor (VIF) ≤ 10), and this model (and its R-squared) would not provide their relative strength of associations with the outcome variables. Significance (alpha) and confidence levels were set at 5 and 95%, respectively.

## Results

### Study and participant characteristics

Characteristics of the three studies included in the analyses are summarised in Additional file 5. Across the three included studies, 7607 (of 8551; 89.0%), 4678 (of 5757; 81.3%), and 2330 (of 2891; 80.6%) participants provided valid accelerometer data at baseline, 1st follow-up and 2nd follow-up, respectively, after excluding unreliable data [17] and data with invalid wear time. Of these, 3871 (of 7607; 50.9%), 2078 (of 4678; 44.4%) and 1024 (of 2330; 43.9%) provided valid data on exposure variables and covariates at baseline, 1st follow-up and 2nd follow-up, respectively, and constituted the samples for cross-sectional (*N* = 3871) and longitudinal (*N* = 2302) analyses. Participants included in the cross-sectional and longitudinal analyses were younger, more likely to have a mother with a high school and university degree, and accumulated less MVPA than those excluded from the analyses (Additional file 6). Regarding domain-specific physical activities (Table 1), approximately 42% of participants actively travelled to school, 90% engaged in organised sports at least occasionally, and over half took part in PE for at least 120 min per week. The average duration of daily MVPA at baseline was (mean ± SD) 54.5 ± 26.0 min in the cross-sectional sample and 54.7 ± 25.6 min in the longitudinal sample, and 35.9 and 36.4% of the samples met current physical activity guidelines, respectively. In the longitudinal sample, monitor wear time increased by 9.9 ± 69.7 min per day, but time spent in MVPA decreased by 6.2 ± 25.5 min per day over 2.5 ± 1.0 years of follow-up across all included studies.

**Table 1:** Descriptive characteristics of participants included in the cross-sectional (*N* = 3871) and longitudinal analyses (*N* = 2302)

	Cross-sectional analysis	Longitudinal analysis
	Frequency (%) or Mean ± SD	Frequency (%) or Mean ± SD
**At baseline**		
Age (year)	11.3 ± 1.2	11.4 ± 1.2
Sex		
*Male*	1801 (46.5)	1048 (45.5)
*Female*	2070 (53.5)	1254 (54.5)
Maternal education		
*High school*	1243 (32.1)	709 (30.8)
*College*	1739 (44.9)	1005 (43.7)
*University*	889 (23.0)	588 (25.5)
Active travel		
*Active mode*	1654 (42.7)	970 (42.1)
*Other mode*	2217 (57.3)	1332 (57.9)
Organised sport		
*Never*	391 (10.1)	240 (10.4)
*Occasionally*	1060 (27.4)	651 (28.3)
*Sometimes*	794 (20.5)	480 (20.9)
*Often*	908 (23.5)	545 (23.7)
*Usually*	718 (18.6)	386 (16.8)
Physical education		
*0–59 min*	207 (5.4)	144 (6.3)
*60–89 min*	546 (14.1)	381 (16.6)
*90–119 min*	949 (24.5)	574 (24.9)
*120–149 min*	1737 (44.9)	952 (41.4)
*≥ 150 min*	432 (11.2)	251 (10.9)
Season		
*Winter*	698 (18.0)	473 (20.6)
*Spring*	1971 (50.9)	1119 (48.6)
*Summer*	717 (18.5)	397 (17.3)
*Autumn*	485 (12.5)	313 (13.6)
MVPA (min/day)	54.5 ± 26.0	54.7 ± 25.6
MPA (min/day)	38.7 ± 16.2	38.6 ± 15.9
VPA (min/day)	15.8 ± 12.6	16.0 ± 12.4
PA guidelines		
*Meeting guidelines*	1391 (35.9)	837 (36.4)
*Not meeting guidelines*	2480 (64.1)	1465 (63.6)
Monitor wear time (min/day)	765.4 ± 60.6	773.4 ± 57.4
**From baseline to follow-ups**^**a**^		
Follow-up duration (year)	–	2.5 ± 1.0
Difference in seasons		
*Same*	–	2052 (66.2)
*Shorter/colder*	–	573 (18.5)
*Longer/warmer*	–	477 (15.4)
Annual change in MVPA (min/day)	–	−2.5 ± 12.6
Annual change in MPA (min/day)	–	−2.2 ± 8.3
Annual change in VPA (min/day)	–	−0.2 ± 6.8
Annual change in monitor wear time (min/day)	–	5.1 ± 37.9
Annual change in number of valid days	–	−0.1 ± 0.6

*MPA* moderate physical activity, *MVPA* moderate-to-vigorous physical activity, *N* number, *PA* physical activity, *SD* standard deviation, *VPA* vigorous physical activity

^a^Data from follow-ups 1 and 2 (3102 observation) were included. Annual change was calculated as: (Change in MVPA/MPA/VPA/wear time) / (Follow-up duration)

### Cross-sectional associations

Participants who actively travelled to school had significantly higher MVPA than those using other travel modes (Table 2; see Additional file 7 for full results including the covariates). Compared to participants who never took part in organised sports, those who ever engaged in organised sports had incrementally greater amounts of MVPA as the frequency increased. The weekly duration of PE between 60 and 89 and ≥ 150 min was positively associated with MVPA, but their coefficients did not enlarge as the duration increased. In secondary analyses, similar patterns of the associations with active travel, organised sport and PE were observed in MPA and VPA.

**Table 2:** Cross-sectional association between domain-specific physical activity and daily accelerometer-assessed MVPA, MPA and VPA (*N* = 3871)

	MVPA				MPA				VPA			
	*Coefficient*	*95% CI*		*P-value*^*†*^	*Coefficient*	*95% CI*		*P-value*^*†*^	*Coefficient*	*95% CI*		*P-value*^*†*^
		*Lower*	*Upper*			*Lower*	*Upper*			*Lower*	*Upper*	
**Active travel** (ref. Other mode)												
Active mode	6.97	5.50	8.45	**< 0.001**	4.62	3.70	5.54	**< 0.001**	2.36	1.61	3.10	**< 0.001**
**Organised sport** (ref. Never)												
Occasionally	3.08	0.42	5.75	**0.023**	2.40	0.73	4.07	**0.005**	0.69	−0.67	2.04	0.321
Sometimes	4.90	2.10	7.70	**0.001**	3.30	1.55	5.06	**< 0.001**	1.59	0.17	3.02	**0.028**
Often	9.23	6.51	11.96	**< 0.001**	6.00	4.29	7.71	**< 0.001**	3.23	1.85	4.61	**< 0.001**
Usually	11.40	8.54	14.26	**< 0.001**	7.31	5.52	9.10	**< 0.001**	4.09	2.64	5.54	**< 0.001**
**Physical education** (ref. 0–59 min)												
60–89 min	4.81	0.81	8.81	**0.019**	2.43	−0.07	4.93	0.057	2.37	0.35	4.39	**0.021**
90–119 min	5.16	1.22	9.11	**0.010**	2.85	0.38	5.32	**0.024**	2.32	0.33	4.31	**0.023**
120–149 min	4.70	0.74	8.66	**0.020**	1.90	−0.58	4.38	0.133	2.80	0.80	4.80	**0.006**
≥ 150 min	6.57	2.26	10.87	**0.003**	3.54	0.85	6.24	**0.010**	3.02	0.85	5.20	**0.006**

The models were adjusted for age, sex, maternal education, season, monitor wear time, and study

*CI* confidence interval, *MPA* moderate physical activity, *MVPA* moderate-to-vigorous physical activity, *VPA* vigorous physical activity

^†^Bold: Significance level at 5%

### Longitudinal associations

In contrast to the cross-sectional associations, baseline active travel, organised sport and PE were not associated with change in MVPA, MPA or VPA (Table 3; see Additional file 8 for full results including the covariates). However, there were trends that the direction of associations were generally positive for active travel and organised sport, despite small coefficients and wide confidence intervals. In particular, those who *often* participated in organised sports at baseline increased MVPA by 3 minutes per day compared to those who never joined organised sports. However, an overall reduction in MVPA annually was observed in both groups (*often*: − 2.3 ± 13.0 min/day [*N* = 731], *never*: − 1.7 ± 10.5 min/day [*N* = 320]; data not shown in Table 1). These contradicting results (i.e., the *often* group had increased MVPA compared to the *never* group, but the *often* had a larger reduction in MVPA annually than the *never*) indicate that the reduction in MVPA might be associated with the covariates included in the model.

**Table 3:** Longitudinal association between domain-specific physical activity and daily accelerometer-assessed MVPA, MPA and VPA (*N* = 2302)

	Change in MVPA				Change in MPA				Change in VPA			
	*Coefficient*	*95% CI*		*P-value*^*†*^	*Coefficient*	*95% CI*		*P-value*^*†*^	*Coefficient*	*95% CI*		*P-value*^*†*^
		*Lower*	*Upper*			*Lower*	*Upper*			*Lower*	*Upper*	
**Active travel** (ref. Other mode)												
Active mode	1.37	−0.28	3.02	0.105	0.84	−0.21	1.88	0.117	0.70	−0.23	1.62	0.140
**Organised sport** (ref. Never)												
Occasionally	0.25	−2.68	3.19	0.866	−0.21	−2.13	1.72	0.832	0.51	−1.12	2.14	0.541
Sometimes	0.28	−2.66	3.23	0.850	0.54	−1.42	2.49	0.589	−0.16	−1.81	1.48	0.845
Often	3.01	−0.12	6.14	0.060	2.02	0.02	4.03	**0.048**	1.17	−0.56	2.91	0.185
Usually	1.41	−1.93	4.74	0.408	1.86	−0.29	4.02	0.091	−0.20	−2.02	1.62	0.831
**Physical education** (ref. 0–59 min)												
60–89 min	0.50	−3.84	4.83	0.823	0.35	−2.29	3.00	0.793	0.24	−2.13	2.62	0.841
90–119 min	−0.05	−4.41	4.31	0.983	0.44	−2.26	3.14	0.750	−0.36	−2.69	1.97	0.763
120–149 min	0.69	−3.64	5.03	0.754	0.79	−1.89	3.46	0.564	0.03	−2.30	2.37	0.977
≥ 150 min	−0.08	−4.73	4.56	0.972	0.27	−2.65	3.19	0.857	−0.25	−2.74	2.24	0.842

The models were adjusted for age, sex, maternal education, change in season, change in monitor wear time, follow-up duration, baseline value of the outcome (MVPA/MPA/VPA) and study

*CI* confidence interval, *MPA* moderate physical activity, *MVPA* moderate-to-vigorous physical activity, *VPA* vigorous physical activity

^†^Bold: Significance level at 5%

### Comparisons between models

To examine which domain of physical activity contributed more to MVPA, standardised exposure variables were computed in both cross-sectional and longitudinal analyses (Table 4; see Additional files 9-10 for full results including the covariates). Cross-sectionally, compared to base models (which included only covariates and explained 21.5% of variance in MVPA), organised sport and active travel additionally explained 1.9 and 1.7% of variance (absolute change), respectively. There were no differences in variance explained between the PE model and the base model. In terms of standardised associations, organised sport generally had the strongest magnitude of associations with MVPA, followed by active travel and PE. PE had a trivial and non-significant association with MVPA (beta coefficient = 0.82; 95% CI: − 0.02, 1.66; *p* = 0.056), but significant association with VPA (beta coefficient = 0.47, 95% CI: 0.05, 0.90; *p* = 0.029) (Additional file 9).

**Table 4:** Comparison of R-squared and standardised coefficients between models with different exposure variables

Model	MVPA		MPA		VPA	
	*R-squared (%)*	*Beta coefficient*^*†*^	*R-squared (%)*	*Beta coefficient*^*†*^	*R-squared (%)*	*Beta coefficient*^*†*^
**Cross-sectional analyses (*N*** **= 3871)**						
Base*	21.5	–	21.4	–	15.2	–
Active travel	23.2	**3.46**	23.4	**2.29**	16.0	**1.17**
Organised sport	23.4	**3.81**	23.4	**2.35**	16.4	**1.46**
Physical education	21.5	0.82	21.5	0.34	15.3	**0.47**
**Longitudinal analyses (*N*** **= 2302)**						
Base*	32.3	–	33.6	–	29.1	–
Active travel	32.4	0.68	33.6	0.41	29.2	0.35
Organised sport	32.4	0.85	33.8	**0.90**	29.1	0.03
Physical education	32.3	0.02	33.6	0.10	29.1	−0.06

*MPA* moderate physical activity, *MVPA* moderate-to-vigorous physical activity, *VPA* vigorous physical activity

*Base models include only covariates

^†^Bold: Significant at *p* < 0.05

In longitudinal analyses, differences in R-squared values between models were very small for MVPA, MPA and VPA. The magnitude of associations with MVPA and MPA followed a similar pattern to the cross-sectional analyses (organised sport > active travel > PE), but active travel had the strongest magnitude with VPA. All these standardised associations, however, were non-significant except the one between organised sport and MPA (beta coefficient = 0.90, 95% CI: 0.35, 1.45; *p* = 0.001) (Additional file 10).

## Discussion

### Summary of main findings

Analyses of this large, heterogeneous dataset showed that cross-sectionally, organised sport, active travel and PE were all positively associated with daily minutes spent in MVPA. Associations were generally stronger for organised sport and active travel than PE, as reflected in both standardised effect sizes and proportion of explained variance. Patterns were similar for MPA and VPA. In longitudinal analyses, none of the examined behaviours predicted change in MVPA, MPA and VPA over the average 2-year follow-up.

### Existing literature on cross-sectional and longitudinal associations

Our findings are consistent with previous research which also reported that active travel [10, 30] and organised sport [31] were cross-sectionally associated with accelerometer-assessed MVPA. Findings from existing research are mixed with regards to the association between PE and MVPA [32]. The current study demonstrated that weekly duration of PE was weakly associated with MVPA. However, a German study showed that children and adolescents aged 6–17 years who participated in PE and organised sports were more likely to meet the physical activity guidelines than those who did not [11]. PE in the UK and Australia is supposed to be a compulsory subject with a minimum of 2 hours per week up to the end of secondary education (age 16) [26, 33]. However, the current study showed that 47.8% of participants reported less than 2 hours of PE per week (Table 1). This disagreement may be because PE is more likely to be marginalised than the other core subjects (e.g., mathematics and languages) for the sake of academic achievement in the education system, particularly in secondary education [33]. Another possible explanation of the weak associations with MVPA may be the majority of PE time could be spent in sedentary (e.g., listening to instructions) [32] or in light activity (e.g., practising skills) rather than accumulating MVPA which may not be the main purpose of PE. Nevertheless, it is important to recognise that PE plays a key role supporting most children and adolescents obtaining fundamental movement skills and confidence to maintain an active life later on [34]. Therefore, the inconsistent findings may depend on the accuracy of reporting (e.g., scheduled or actual PE duration reported by teacher, child or parent) and the amount of MVPA allocated in a PE class.

We found that none of the physical activity domains predicted change in MVPA. Previous research has also shown a lack of longitudinal associations between active travel, organised sport and PE and change in accelerometer-assessed MVPA. A study with Norwegian children reported no associations between active travel or organised sport and change in MVPA from age nine to 15 years [35]. No previous studies have examined the association between multiple behaviours at baseline and change in device-assessed MVPA. Brooke et al. [36] showed that neither baseline weekly physical activity variety (i.e., the number of different activities) or frequency (i.e., number of activity sessions) nor changes in variety and frequency were associated with change in MVPA. These findings consistently show that domain-specific activity participation at baseline does not predict change in MVPA, and even change in activity participation might not be associated with change in MVPA. Given that data on physical activity domains were self-reported, the accuracy of reporting particularly with high level of granularity (e.g., number, frequency, duration) might be warranted. Further development in the accuracy of self-reported (the number, frequency, duration of) physical activity domains will be required.

Can we therefore predict future MVPA using domain-specific physical activity (e.g., active travel, organised sport and PE) during childhood? Given the average decline and change in the modes of activity during childhood and adolescence [37] as well as changes in other factors (e.g., individual, social, environmental) associated with these behavioural changes, long-term prediction is highly challenging compared to establishing cross-sectional associations. For example, changes in travel behaviour often occur during the transition between schools (e.g., from primary to secondary school), which tends to be related to distance to school, safety and independent mobility [38–40]. Werneck et al. [41] studied the association between change in active travel to school and change in MPA and VPA using the same ICAD dataset (six longitudinal studies including ALSPAC, CLAN and SPEEDY). They found that, compared to adolescents (aged 10–13 years) maintaining active travel, those who maintained or changed to passive travel showed large decreases in MPA and VPA. In contrast, those who took up active travel showed smaller decreases in VPA compared to those maintaining active travel. Some sports, such as football and dance, are still popular during adolescence, whereas participation in gymnastics, for example, is high during childhood but tends to wane during adolescence [36, 42]. As most participants included in the current longitudinal analyses experienced school transitions (i.e., from primary or secondary to secondary or senior secondary) and developmental transitions (i.e., from childhood to adolescence), the absence of longitudinal associations might be partially because we modelled baseline physical activity domain data as the exposures. Unfortunately, change in domain-specific physical activity participation was not available for the studies included in this analysis. Thus, future research examining longitudinal associations between changes in domain-specific physical activity and changes in device-assessed MVPA may be worthwhile.

The current study showed that organised sport, active travel and PE independently accounted for less than 2% of the variance in MVPA, in addition to the base model. This finding indicates that active travel, organised sport and PE may not be major contributing factors to MVPA, but the potential impact of methodological and analytical issues should be also considered. For example, active travel was only captured in the context of ‘to school’, and activities included in organised sport were diverse in terms of their type and number because they were retrospectively determined and harmonised on the basis of our definition. MVPA was measured on weekdays and over the weekend, but active travel to school and PE occurred mainly on weekdays. Furthermore, it is uncertain whether interactions between the examined behaviours (e.g., those who actively travelled to school were more likely to participate in organised sport) might impact MVPA. Despite these caveats, the lack of associations suggest that a wide range of domain-specific physical activity should be promoted to accumulate sufficient MVPA and to minimise the age-related decline in MVPA during childhood and adolescence. Creating supportive environments (including schools and social and built environment) and providing enough opportunities and grassroots support may enable young people, and particularly adolescents, to maintain and/or improve MVPA in the longer term [13, 43].

### Strengths and limitations

The current study applied novel harmonisation and analytical approaches to examine how different domain-specific physical activities contributed to MVPA cross-sectionally and longitudinally. We focused on policy-relevant physical activity domains with the potential for investments and interventions, and used accelerometer-assessed MVPA as well as MPA and VPA separately. Covariates were carefully selected based on the literature. Furthermore, we included a relatively heterogeneous and large sample size. Despite these strengths, this study has limitations. Three domains of physical activity might not capture the full breadth of activity behaviours. In relation to school, for example, other classes (than PE), recess and before- and after-school programmes can provide opportunities to be physically active [32, 44]. Household chores and active play (e.g., games and play in the playground, street or backyard) can also contribute to daily MVPA [45]. No data were available in ICAD to include these behaviours in the current analysis, but they may be worth of study in future research. In the process of harmonisation, we made effort to minimise self-reported bias (e.g., prioritising teacher over child reports for PE). Although self-reports have been the most feasible approach to collect domain-specific physical activity, potentially erroneous data were unavoidable due to the nature of self-reports. To overcome this issue, we used categorical rather than continuous variables to adjust subjective assessment of frequency and duration of activities. The data were collected between 2003 and 2011. While there may have been secular changes in the prevalence of behaviours, we consider it unlikely that these will have impacted on the associations studied here. While the ICAD project includes data from more countries, only three studies (one from Australia and two from England) collected the necessary data to be included in the current analyses.

### Implications and future recommendations

Interventions and policy actions should support a multi-sectoral approach where sports, transport, urban design, and civic and private organisations synergise with each other [46]. Our findings suggest that participation in organised sport and active travel may have potential positive implications for MVPA in children and adolescent, but that participation at a single time point is not predictive for future activity levels. Accordingly, it is important to provide all children and adolescents with safe, equitable and varied opportunities to be active. Collecting consistent and repeated measures of domain-specific physical activity is also critical to further our understanding of how physical activity accumulated in different domains impacts on overall activity levels. In the ICAD project, only two (i.e., CLAN and SPEEDY) of 23 studies simultaneously measured active travel, organised sport (frequency) and PE (duration) at multiple time points. To advance the current knowledge, longitudinal studies which measure domain-specific physical activity (including its type, frequency and duration) and device-based physical activity simultaneously and on repeated occasions are required.

## Conclusions

Among children and adolescents, their participation in organised sport and active travel contributed more to MVPA than PE cross-sectionally. Longitudinally, however, none of the physical activity domains predicted change in MVPA. A multi-sectoral approach covering a wide range of physical activity domains should be promoted to minimise the age-related decline in MVPA during childhood.

## Supplementary Information

**Additional file 1.**

**Additional file 2.**

**Additional file 3.**

**Additional file 4.**

**Additional file 5.**

**Additional file 6.**

**Additional file 7.**

**Additional file 8.**

**Additional file 9.**

**Additional file 10.**

## Data Availability

The original data request and pre-specified analysis plan are available from the corresponding author on reasonable request. De-identified participant data can be also made available upon reasonable request, following standard ICAD data request procedures (see for information: http://www.mrc-epid.cam.ac.uk/research/studies/icad/).

## Abbreviations

ALSPAC: Avon Longitudinal Study of Parents and Children
CLAN: Children Living in Active Neighbourhoods
ICAD: International Children’s Accelerometry Database
MPA: Moderate physical activity
MVPA: Moderate-to-vigorous physical activity
PE: Physical education
SPEEDY: Sport, Physical activity and Eating behaviour: Environmental Determinants in Young people
VPA: Vigorous physical activity
