# Supplementary material for: Cross-sectional and longitudinal associations of active travel, organised sport and physical education with accelerometer-assessed moderate-to-vigorous physical activity in young people: the International Children’s Accelerometry Database
Source: Int J Behav Nutr Phys Act. 2022 Apr 2;19:41. doi: 10.1186/s12966-022-01282-4 (PMC8977036; doi:10.1186/s12966-022-01282-4)
Supplement: Supplementary file 1 — Additional file 1. [file 12966_2022_1282_MOESM1_ESM.docx]

# Additional File 1

## STrengthening the Reporting of OBservational studies in Epidemiology (STROBE) guidelines for observational studies

|  | **Item No.** | | **Recommendation** | | **Page  No.** | | | **Relevant text from manuscript** |
| --- | --- | --- | --- | --- | --- | --- | --- | --- |
| **Title and abstract** | 1 | | (*a*) Indicate the study’s design with a commonly used term in the title or the abstract | | n/a | | | “Cross-sectional and longitudinal associations…” |
|  |  |  | (*b*) Provide in the abstract an informative and balanced summary of what was done and what was found | | n/a | | | See Abstract |
| **Introduction** | | | | | | | |  |
| Background/rationale | 2 | | Explain the scientific background and rationale for the investigation being reported | | p. 1 | | | See Background |
| Objectives | 3 | | State specific objectives, including any prespecified hypotheses | | p.p. 1-2 | | | See Background |
| **Methods** | | | | | | | |  |
| Study design | 4 | | Present key elements of study design early in the paper | | p. 2 | | | See Methods > Study selection and participants |
| Setting | 5 | | Describe the setting, locations, and relevant dates, including periods of recruitment, exposure, follow-up, and data collection | | p. 2 | | | See Methods > Study selection and participants |
| Participants | 6 | | (*a*) *Cohort study*—Give the eligibility criteria, and the sources and methods of selection of participants. Describe methods of follow-up  *Case-control study*—Give the eligibility criteria, and the sources and methods of case ascertainment and control selection. Give the rationale for the choice of cases and controls  *Cross-sectional study*—Give the eligibility criteria, and the sources and methods of selection of participants | | p. 2 | | | See Methods > Study selection and participants |
|  |  |  | (*b*) *Cohort study*—For matched studies, give matching criteria and number of exposed and unexposed  *Case-control study*—For matched studies, give matching criteria and the number of controls per case | | n/a | | | n/a |
| Variables | 7 | | Clearly define all outcomes, exposures, predictors, potential confounders, and effect modifiers. Give diagnostic criteria, if applicable | | p.p. 2-5 | | | See Methods > Measures |
| Data sources/ measurement | 8* | | For each variable of interest, give sources of data and details of methods of assessment (measurement). Describe comparability of assessment methods if there is more than one group | | p.p. 2-5 | | | See Methods > Measures; and Additional Files 2-4 |
| Bias | 9 | | Describe any efforts to address potential sources of bias | | p.p. 2-5 | | | See Methods > Measures; and Additional Files 2-4 |
| Study size | 10 | | Explain how the study size was arrived at | | p. 5 | | | See Methods > Data analyses |
| Quantitative variables | 11 | | Explain how quantitative variables were handled in the analyses. If applicable, describe which groupings were chosen and why | p.p. 2-5 | | | | See Methods > Measures |
| Statistical methods | 12 | | (*a*) Describe all statistical methods, including those used to control for confounding | p.p. 5 | | | | See Methods > Data analyses |
|  |  | | (*b*) Describe any methods used to examine subgroups and interactions | n/a | | | | n/a |
|  |  | | (*c*) Explain how missing data were addressed | p. 5 | | | | See Methods > Data analyses |
|  |  |  | (*d*) *Cohort study*—If applicable, explain how loss to follow-up was addressed  *Case-control study*—If applicable, explain how matching of cases and controls was addressed  *Cross-sectional study*—If applicable, describe analytical methods taking account of sampling strategy | p.p. 5 | | | | See Methods > Data analyses |
|  |  |  | (*e*) Describe any sensitivity analyses | n/a | | | | n/a |
| **Results** |  | | | | | | | |
| Participants | 13* | | (a) Report numbers of individuals at each stage of study—eg numbers potentially eligible, examined for eligibility, confirmed eligible, included in the study, completing follow-up, and analysed | p. 6 | | | | See Results > Study and participant characteristics |
|  |  |  | (b) Give reasons for non-participation at each stage | p. 6 | | | | See Results > Study and participant characteristics |
|  |  |  | (c) Consider use of a flow diagram | n/a | | | | n/a |
| Descriptive data | 14* | | (a) Give characteristics of study participants (eg demographic, clinical, social) and information on exposures and potential confounders | p. 6 | | | | See Results > Study and participant characteristics; and Table 1 |
|  |  |  | (b) Indicate number of participants with missing data for each variable of interest | n/a | | | | Additional File 6 |
|  |  |  | (c) *Cohort study*—Summarise follow-up time (eg, average and total amount) | p. 6 | | | | See Results > Study and participant characteristics; and Table 1 |
| Outcome data | 15* | | *Cohort study*—Report numbers of outcome events or summary measures over time | n/a | | | | n/a |
|  |  |  | *Case-control study—*Report numbers in each exposure category, or summary measures of exposure | n/a | | | | n/a |
|  |  |  | *Cross-sectional study—*Report numbers of outcome events or summary measures | n/a | | | | See Table 1 |
| Main results | 16 | | (*a*) Give unadjusted estimates and, if applicable, confounder-adjusted estimates and their precision (eg, 95% confidence interval). Make clear which confounders were adjusted for and why they were included | p.p. 6-8 | | | | See Results > Cross-sectional associations, Table 2, Additional File 7; Results > Longitudinal associations, Table 3, Additional File 8; Results > Comparisons between models, Table 4, Additional Files 9-10;  (Confounders were listed and explained in Methods > Data analyses; and Discussion > Strengths and limitations) |
|  |  |  | (*b*) Report category boundaries when continuous variables were categorized | p.p. 2-5 | | | | See Methods > Measures; and Additional Files 3-4 |
|  |  |  | (*c*) If relevant, consider translating estimates of relative risk into absolute risk for a meaningful time period | n/a | | | | n/a |
| Other analyses | 17 | Report other analyses done—eg analyses of subgroups and interactions, and sensitivity analyses | | | | n/a | n/a | |
| **Discussion** | | | | | | | | |
| Key results | 18 | Summarise key results with reference to study objectives | | | | p. 8 | See Discussion > Summary of main findings | |
| Limitations | 19 | Discuss limitations of the study, taking into account sources of potential bias or imprecision. Discuss both direction and magnitude of any potential bias | | | | p.p. 10-11 | See Discussion > Strengths and limitations | |
| Interpretation | 20 | Give a cautious overall interpretation of results considering objectives, limitations, multiplicity of analyses, results from similar studies, and other relevant evidence | | | | p.p. 8-10 | See Discussion > Existent literature on cross-sectional and longitudinal associations | |
| Generalisability | 21 | Discuss the generalisability (external validity) of the study results | | | | p.p. 10-11 | See Discussion > Strengths and limitations | |
| **Other information** | |  | | | | | | |
| Funding | 22 | Give the source of funding and the role of the funders for the present study and, if applicable, for the original study on which the present article is based | | | | p.p. 14 | See Declarations > Funding | |

*Give information separately for cases and controls in case-control studies and, if applicable, for exposed and unexposed groups in cohort and cross-sectional studies.

**Note:** An Explanation and Elaboration article discusses each checklist item and gives methodological background and published examples of transparent reporting. The STROBE checklist is best used in conjunction with this article (freely available on the Web sites of PLoS Medicine at http://www.plosmedicine.org/, Annals of Internal Medicine at http://www.annals.org/, and Epidemiology at http://www.epidem.com/). Information on the STROBE Initiative is available at www.strobe-statement.org.
