# Supplementary material for: Cross-sectional and longitudinal associations of active travel, organised sport and physical education with accelerometer-assessed moderate-to-vigorous physical activity in young people: the International Children’s Accelerometry Database
Source: Int J Behav Nutr Phys Act. 2022 Apr 2;19:41. doi: 10.1186/s12966-022-01282-4 (PMC8977036; doi:10.1186/s12966-022-01282-4)
Supplement: Supplementary file 2 — Additional file 2. [file 12966_2022_1282_MOESM2_ESM.docx]

# Additional File 2

## Harmonisation processes: Active travel

The harmonisation of this variable was documented and shared on the ICAD webpage (www.mrc-epid.cam.ac.uk/research/studies/icad/data-harmonisation/) under the name of ‘School travel’ (in the category of *Physical activity – behaviour and correlates*).

### ALSPAC

The questionnaire allowed for selection of multiple modes of travel to school and the frequency of each travel mode including ‘every/most days’ and ‘some days’. When participants reported multiple travel modes and their frequencies, the most frequently used mode of travel (i.e., ‘every/most days’) was included in the harmonisation variable. If there were more than one ‘every/most days’ responses, the following priority order was applied: *car*, *public transport*, *cycle*, *walk*, and *other*. The same process was also used for multiple ‘some days’ responses and associated travel modes.

### CLAN

The questionnaire allowed for selection of multiple modes of travel to school. If more than one travel modes were reported, the most frequently used mode of travel was selected for the harmonised variable. When there were more than one travel modes with the same frequency were reported, the following priority order was applied: *car*, *public transport*, *cycle*, *walk*, and *other*.

### SPEEDY

The questionnaire asked for the duration and frequency of walking and cycling to school, and the combined duration and frequency of travelling to school by bus or car.
