# Supplementary material for: Cross-sectional and longitudinal associations of active travel, organised sport and physical education with accelerometer-assessed moderate-to-vigorous physical activity in young people: the International Children’s Accelerometry Database
Source: Int J Behav Nutr Phys Act. 2022 Apr 2;19:41. doi: 10.1186/s12966-022-01282-4 (PMC8977036; doi:10.1186/s12966-022-01282-4)
Supplement: Supplementary file 3 — Additional file 3. [file 12966_2022_1282_MOESM3_ESM.docx]

# Additional File 3

## Harmonisation and categorisation processes: Organised sport

The harmonisation of this variable was documented and shared on the ICAD webpage (www.mrc-epid.cam.ac.uk/research/studies/icad/data-harmonisation/) under the name of ‘Organised sport’ (in the category of *Physical activity – behaviour and correlates*).

One of the challenges that we faced when harmonising the weekly frequency of organised sports was that each study measured different types and numbers of organised sports., The frequency was calculated by summing the scores from each organised sport item (ranging from 0-59.5 times/week).

### ALSPAC

Two types of organised sports were included: *swimming* and *sport class*. Categorical responses were converted to scores: ‘not at all’ or ‘less than once a month’ = 0, ‘1-3 times per month’ = 0.5, ‘once a week’ = 1, ‘2-5 times per week’ = 3.5, and ‘nearly every day’ = 6.

### CLAN

The frequency of organised sports was reported separately on weekdays (from Monday to Friday) and at weekends (Saturday and Sunday). In the harmonised variable, the weekday and weekend data were merged to create a weekly frequency (times/week). Twenty types of organised sports were included: *Aussie rules football*, *baseball*, *basketball*, *cricket*, *dance*, *gymnastics*, *netball*, *soccer*, *school sport class*, *tennis*, *golf*, *hockey*, *martial arts*, *rowing*, *rugby*, *squash*, *surfing*, *touch football*, *volleyball*, and *swimming*. A response to each organised sport item was capped at seven (ranging from 0-7 times/week).

### SPEEDY

Fourteen types of organised sports were included: *softball/rounders*, *basketball/volleyball*, *cricket*, *dance*, *football*, *gymnastics*, *hockey*, *martial arts*, *netball*, *rugby*, *tennis/badminton/squash*, *bowling*, *skiing/snowboarding/sledging*, *swimming*. Categorical responses were converted to scores using the same scoring system as this study: ‘never’ = 0, ‘once’ = 1, ‘2-3 times’ = 2.5, and ‘more than 4 times’ = 4.5.

Based on zero and quartile values (25th, 50th, 75th) of each study, the frequency was then classified as ‘never’, ‘occasionally’, ‘sometimes’, ‘often’ and ‘usually’. For SPEEDY, missing values were imputed using a modal value of two (times per week) if data on the session length were reported as > 0 minutes.

|  |  | Percentiles | | |  | Category | | | | |
| --- | --- | --- | --- | --- | --- | --- | --- | --- | --- | --- |
|  | N | 25th | 50th | 75th |  | Never | Occasionally | Sometimes | Often | Usually |
| ALSPAC | 3,903 | 1 | 2 | 4 |  | 0 | 0.5-1 | 1.5-2 | 2.5-4 | >4 |
| CLAN | 435 | 4 | 6 | 10 |  | 0 | 0.5-4 | 4.5-6 | 6.5-10 | >10 |
| SPEEDY | 1,583 | 3 | 6 | 11 |  | 0 | 0.5-3 | 3.5-6 | 6.5-11 | >11 |
