# Supplementary material for: Cross-sectional and longitudinal associations of active travel, organised sport and physical education with accelerometer-assessed moderate-to-vigorous physical activity in young people: the International Children’s Accelerometry Database
Source: Int J Behav Nutr Phys Act. 2022 Apr 2;19:41. doi: 10.1186/s12966-022-01282-4 (PMC8977036; doi:10.1186/s12966-022-01282-4)
Supplement: Supplementary file 4 — Additional file 4. [file 12966_2022_1282_MOESM4_ESM.docx]

# Additional File 4

## Harmonisation and categorisation processes: Physical education

The harmonisation of this variable was documented and shared on the ICAD webpage (www.mrc-epid.cam.ac.uk/research/studies/icad/data-harmonisation/) under the name of ‘Physical education’ (in the category of *Physical activity – behaviour and correlates*).

### ALSPAC

The questionnaire asked for the duration of physical education by hour and minute (e.g., 1 hour and 30 minutes). The hour data were converted to minutes and added to the minute data to generate a total duration variable (e.g., 90 minutes).

### CLAN

The duration of physical education (in minutes) was reported separately on weekdays (from Monday to Friday) and at weekends (Saturday and Sunday). In the harmonised variable, the weekday and weekend data were merged to create a weekly duration (minutes/week).

### SPEEDY

The questionnaire asked for the duration of physical education by hour. The hour data were converted to minutes.

The harmonised data were not normally distributed. Just under 40% of participants reported 120 minutes per week (39.2%), followed by 90 minutes (17.4%), 60 minutes (9.6%) and 150 minutes (7.7%). Therefore, the data (range: 0-1200 minutes/week) were categorised into five groups (i.e., ‘0-59’, ‘60-89’, ‘90-119’, ‘120-149’, and ‘≥150’) based on these cutoffs and the Governments’ recommendations of time spent on physical education per week^1^.

^1^According to the Education Act 2002, the England government does not set a specific target for how much curriculum time schools must dedicate to physical education [1]. However, in February 2013, the Office for Standards in Education (Ofsted) recommended that primary schools should spend at least 120 minutes a week of physical education [1]. Department for Education proposed the healthy schools rating scheme as part of the Childhood Obesity Plan [2]. The rating scheme includes time spent on physical education as well as the promotion of active travel, food education, and compliance with the school food standards. Schools receive a certificate if they achieve Gold, Silver or Bronze awards. The scoring system of time spent on physical education in schools are:

- < 90 minutes per week = 0 point
- 90-119 minutes per week = 15 points (required for Bronze)
- 120-149 minutes per week = 25 points (required for Silver/Gold)
- ≤ 150 minutes per week = 35 points

According to the State Government of Victoria in Australia [3], all Victorian government schools are required to meet the following amount of physical and sport education* at minimum:

- Year 4 to Year 6 (Primary School; ages 9-12 years): 90 minutes per week
- Year 7 to Year 10 (Secondary School; ages 12-16): 100 minutes per week

*Physical education is defined as “the delivery of the physical education curriculum through timetabled and structured classes”. Sport is defined as “any form of sport (inter or intra school) within the educational setting which is timetabled into the school week”.

# References

1. Foster D, Roberts N: Physical education, physical activity and sport in schools. In*.*: UK Parliament; 2019: 1-28.

2. Department for Education. Healthy schools rating scheme: Guidance for schools. 2019. https://assets.publishing.service.gov.uk/government/uploads/system/uploads/attachment_data/file/906875/Healthy_schools_rating_scheme.pdf.

3. Physical and sport education - delivery requirements. https://www2.education.vic.gov.au/pal/physical-and-sport-education-delivery-requirements/policy. Accessed May 27 2021.
