# Supplementary material for: Cross-sectional and longitudinal associations of active travel, organised sport and physical education with accelerometer-assessed moderate-to-vigorous physical activity in young people: the International Children’s Accelerometry Database
Source: Int J Behav Nutr Phys Act. 2022 Apr 2;19:41. doi: 10.1186/s12966-022-01282-4 (PMC8977036; doi:10.1186/s12966-022-01282-4)
Supplement: Supplementary file 5 — Additional file 5. [file 12966_2022_1282_MOESM5_ESM.docx]

# Additional File 5

## Characteristics of three studies and their participants at baseline and follow-ups

|  |  | **ALSPAC** | **CLAN*** | **SPEEDY** |
| --- | --- | --- | --- | --- |
| Country (City) |  | England (Bristol) | Australia (Melbourne) | England (Norfolk) |
| Year | Baseline | 2003 | 2004 | 2007 |
|  | Follow-up 1 | 2005 | 2006 | 2008 |
|  | Follow-up 2 | 2007 | - | 2011 |
| Sample size^‡^ | Baseline | 2109 (54.5%) | 405 (10.5%) | 1357 (35.1%) |
|  | Follow-up 1 | 1248 (60.1%) | 291 (14.0%) | 539 (25.9%) |
|  | Follow-up 2 | 762 (74.4%) | 0 (0.0%) | 262 (25.6%) |
| Age (year)^‡^ | Baseline | 11.8 ± 0.2 | 12.4 ± 2.7 | 10.3 ± 0.3 |
|  | Follow-up 1 | 13.9 ± 0.2 | 14.0 ± 2.7 | 11.2 ± 0.3 |
|  | Follow-up 2 | 15.5 ± 0.2 | - | 14.3 ± 0.3 |

*The first wave was in 2001 (age: 10.19 ± 2.37).

^‡^Sample size (Baseline: N = 3871, Follow-up 1: N = 2078, Follow-up 2: N = 1024) and age were based on observations included in models.

ALSPAC = Avon Longitudinal Study of Parents and Children [4, 5]; CLAN = Children Living in Active Neighbourhoods (originally named as the Children's Leisure Activities Study [CLASS]) [6]; N = number; SPEEDY = Sport, Physical activity and Eating behaviour: Environmental Determinants in Young people [7].

# References

4. Boyd A, Golding J, Macleod J, Lawlor DA, Fraser A, Henderson J, Molloy L, Ness A, Ring S, Davey Smith G. Cohort profile: The ‘Children of the 90s’—the index offspring of the Avon Longitudinal Study of Parents and Children. Int J Epidemiol. 2013; doi:10.1093/ije/dys064.

5. Fraser A, Macdonald-Wallis C, Tilling K, Boyd A, Golding J, Davey Smith G, Henderson J, Macleod J, Molloy L, Ness A *et al*. Cohort Profile: The Avon Longitudinal Study of Parents and Children: ALSPAC mothers cohort. Int J Epidemiol. 2012; doi:10.1093/ije/dys066.

6. Telford A, Salmon J, Timperio A, Crawford D. Quantifying and characterizing physical activity among 5- to 6- and 10- to 12-year-old children: The Children’s Leisure Activities Study (CLASS). Pediatr Exerc Sci. 2005; doi:10.1123/pes.17.3.266.

7. van Sluijs EMF, Skidmore PML, Mwanza K, Jones AP, Callaghan AM, Ekelund U, Harrison F, Harvey I, Panter J, Wareham NJ *et al*. Physical activity and dietary behaviour in a population-based sample of British 10-year old children: the SPEEDY study (Sport, Physical activity and Eating behaviour: Environmental Determinants in Young people). BMC Public Health. 2008; doi:10.1186/1471-2458-8-388.
