# Supplementary material for: Cross-sectional and longitudinal associations of active travel, organised sport and physical education with accelerometer-assessed moderate-to-vigorous physical activity in young people: the International Children’s Accelerometry Database
Source: Int J Behav Nutr Phys Act. 2022 Apr 2;19:41. doi: 10.1186/s12966-022-01282-4 (PMC8977036; doi:10.1186/s12966-022-01282-4)
Supplement: Supplementary file 6 — Additional file 6. [file 12966_2022_1282_MOESM6_ESM.docx]

# Additional File 6

## Baseline data of participants included and excluded from cross-sectional and longitudinal analyses

|  | **Cross-sectional analysis** | | | |  |  | **Longitudinal analysis** | | | |  |
| --- | --- | --- | --- | --- | --- | --- | --- | --- | --- | --- | --- |
|  | Included | | Excluded | |  |  | Included | | Excluded | |  |
|  | N | % or Mean±SD | N | % or Mean±SD | Difference* |  | N | % or Mean±SD | N | % or Mean±SD | Difference* |
| Age (year) | 3871 | 11.3±1.2 | 4663 | 11.7±0.8 | *t* = -15.37 (*p*<0.001) |  | 2302 | 11.4±1.2 | 6232 | 11.5±0.9 | *t* = -5.11 (*p*<0.001) |
| Sex (female) | 3871 | 53.5 | 4666 | 52.7 | *X^2^* = 0.54 (*p*=0.463) |  | 2302 | 54.5 | 6235 | 52.5 | *X^2^* = 2.60 (*p*=0.107) |
| Maternal education (high school; college; university) | 3871 | 32.1; 44.9; 23.0 | 3030 | 30.8; 43.7; 25.5 | *X^2^* = 44.10 (*p*<0.001) |  | 2302 | 30.8; 43.7; 25.5 | 4599 | 28.0; 49.0; 23.1 | *X^2^* = 17.36 (*p*<0.001) |
| MVPA (min/day) | 3871 | 54.5±26.0 | 4680 | 58.1±58.5 | *t* = -3.58 (*p*<0.001) |  | 2302 | 54.7±25.6 | 6249 | 57.1±52.4 | *t* = -2.17 (*p*=0.030) |

*Differences were tested using either independent t-tests or chi-squared (*X*^2^) tests
