# Supplementary material for: Cross-sectional and longitudinal associations of active travel, organised sport and physical education with accelerometer-assessed moderate-to-vigorous physical activity in young people: the International Children’s Accelerometry Database
Source: Int J Behav Nutr Phys Act. 2022 Apr 2;19:41. doi: 10.1186/s12966-022-01282-4 (PMC8977036; doi:10.1186/s12966-022-01282-4)
Supplement: Supplementary file 7 — Additional file 7. [file 12966_2022_1282_MOESM7_ESM.docx]

# Additional File 7

## Cross-sectional association of domain-specific physical activity and covariates with daily accelerometer-assessed MVPA, MPA and VPA (N = 3871)

|  | **MVPA** | | | |  | **MPA** | | | |  | **VPA** | | | |
| --- | --- | --- | --- | --- | --- | --- | --- | --- | --- | --- | --- | --- | --- | --- |
|  | *Coefficient* | *95% CI* | | *P-value^†^* |  | *Coefficient* | *95% CI* | | *P-value^†^* |  | *Coefficient* | *95% CI* | | *P-value^†^* |
|  |  | *Lower* | *Upper* |  |  |  | *Lower* | *Upper* |  |  |  | *Lower* | *Upper* |  |
| **Active travel** (ref. Other mode) | | | | | | | | | | | | | | |
| Active mode | 6.97 | 5.50 | 8.45 | **<0.001** |  | 4.62 | 3.70 | 5.54 | **<0.001** |  | 2.36 | 1.61 | 3.10 | **<0.001** |
| Study (ref. SPEEDY) | | | | | | | | | | | | | | |
| ALSPAC | 12.48 | 10.30 | 14.67 | **<0.001** |  | 5.97 | 4.60 | 7.33 | **<0.001** |  | 6.51 | 5.40 | 7.62 | **<0.001** |
| CLAN | 20.04 | 16.80 | 23.27 | **<0.001** |  | 7.51 | 5.49 | 9.53 | **<0.001** |  | 12.53 | 10.88 | 14.17 | **<0.001** |
| Age | -2.64 | -3.45 | -1.82 | **<0.001** |  | -1.60 | -2.11 | -1.08 | **<0.001** |  | -1.04 | -1.46 | -0.63 | **<0.001** |
| Sex (ref. Male) | | | | | | | | | | | | | | |
| Female | -19.39 | -20.83 | -17.94 | **<0.001** |  | -12.94 | -13.84 | -12.03 | **<0.001** |  | -6.45 | -7.18 | -5.71 | **<0.001** |
| Maternal education (ref. High school) | | | | | | | | | | | | | | |
| College | -1.58 | -3.30 | 0.13 | 0.071 |  | -0.74 | -1.81 | 0.34 | 0.178 |  | -0.85 | -1.72 | 0.03 | 0.057 |
| University | -1.62 | -3.62 | 0.37 | 0.111 |  | -1.14 | -2.38 | 0.11 | 0.075 |  | -0.49 | -1.50 | 0.53 | 0.347 |
| Season (ref. Winter) | | | | | | | | | | | | | | |
| Spring | 6.79 | 4.55 | 9.02 | **<0.001** |  | 4.37 | 2.98 | 5.77 | **<0.001** |  | 2.42 | 1.28 | 3.55 | **<0.001** |
| Summer | 5.29 | 2.77 | 7.80 | **<0.001** |  | 3.68 | 2.11 | 5.25 | **<0.001** |  | 1.60 | 0.33 | 2.88 | **0.014** |
| Autumn | -1.02 | -3.75 | 1.71 | 0.465 |  | -0.75 | -2.46 | 0.96 | 0.389 |  | -0.27 | -1.66 | 1.12 | 0.704 |
| Monitor wear time (min/day) | 0.06 | 0.05 | 0.07 | **<0.001** |  | 0.04 | 0.03 | 0.04 | **<0.001** |  | 0.02 | 0.02 | 0.03 | **<0.001** |
| Constant | 34.77 | 22.40 | 47.15 | **<0.001** |  | 27.37 | 19.63 | 35.10 | **<0.001** |  | 7.41 | 1.12 | 13.69 | **0.021** |
| **Organised sport** (ref. Never) | |  | | | | |  | | | | |  | | |
| Occasionally | 3.08 | 0.42 | 5.75 | **0.023** |  | 2.40 | 0.73 | 4.07 | **0.005** |  | 0.69 | -0.67 | 2.04 | 0.321 |
| Sometimes | 4.90 | 2.10 | 7.70 | **0.001** |  | 3.30 | 1.55 | 5.06 | **<0.001** |  | 1.59 | 0.17 | 3.02 | **0.028** |
| Often | 9.23 | 6.51 | 11.96 | **<0.001** |  | 6.00 | 4.29 | 7.71 | **<0.001** |  | 3.23 | 1.85 | 4.61 | **<0.001** |
| Usually | 11.40 | 8.54 | 14.26 | **<0.001** |  | 7.31 | 5.52 | 9.10 | **<0.001** |  | 4.09 | 2.64 | 5.54 | **<0.001** |
| Study (ref. SPEEDY) | | | | | | | | | | | | | | |
| ALSPAC | 13.74 | 11.54 | 15.94 | **<0.001** |  | 6.75 | 5.37 | 8.13 | **<0.001** |  | 6.99 | 5.88 | 8.11 | **<0.001** |
| CLAN | 19.34 | 16.12 | 22.57 | **<0.001** |  | 6.99 | 4.97 | 9.01 | **<0.001** |  | 12.35 | 10.72 | 13.99 | **<0.001** |
| Age | -2.97 | -3.79 | -2.15 | **<0.001** |  | -1.80 | -2.31 | -1.29 | **<0.001** |  | -1.17 | -1.59 | -0.75 | **<0.001** |
| Sex (ref. Male) | | | | | | | | | | | | | | |
| Female | -18.80 | -20.26 | -17.35 | **<0.001** |  | -12.60 | -13.51 | -11.69 | **<0.001** |  | -6.21 | -6.95 | -5.47 | **<0.001** |
| Maternal education (ref. High school) | | | | | | | | | | | | | | |
| College | -2.04 | -3.76 | -0.33 | **0.020** |  | -1.04 | -2.12 | 0.03 | 0.057 |  | -1.00 | -1.87 | -0.13 | **0.025** |
| University | -2.81 | -4.80 | -0.81 | **0.006** |  | -1.92 | -3.17 | -0.67 | **0.003** |  | -0.88 | -1.90 | 0.13 | 0.087 |
| Season (ref. Winter) | |  | | | | |  | | | | |  | | |
| Spring | 6.67 | 4.44 | 8.91 | **<0.001** |  | 4.30 | 2.90 | 5.70 | **<0.001** |  | 2.37 | 1.24 | 3.51 | **<0.001** |
| Summer | 5.27 | 2.76 | 7.79 | **<0.001** |  | 3.67 | 2.10 | 5.24 | **<0.001** |  | 1.60 | 0.33 | 2.88 | **0.014** |
| Autumn | -1.12 | -3.85 | 1.61 | 0.421 |  | -0.82 | -2.53 | 0.88 | 0.344 |  | -0.30 | -1.68 | 1.09 | 0.674 |
| Monitor wear time (min/day) | 0.06 | 0.05 | 0.07 | **<0.001** |  | 0.04 | 0.03 | 0.05 | **<0.001** |  | 0.02 | 0.02 | 0.03 | **<0.001** |
| Constant | 33.85 | 21.34 | 46.36 | **<0.001** |  | 26.67 | 18.83 | 34.50 | **<0.001** |  | 7.18 | 0.83 | 13.53 | **0.027** |
| **Physical education** (ref. 0-59 min) | | | | | | | | | | | | | | |
| 60-89 min | 4.81 | 0.81 | 8.81 | **0.019** |  | 2.43 | -0.07 | 4.93 | 0.057 |  | 2.37 | 0.35 | 4.39 | **0.021** |
| 90-119 min | 5.16 | 1.22 | 9.11 | **0.010** |  | 2.85 | 0.38 | 5.32 | **0.024** |  | 2.32 | 0.33 | 4.31 | **0.023** |
| 120-149 min | 4.70 | 0.74 | 8.66 | **0.020** |  | 1.90 | -0.58 | 4.38 | 0.133 |  | 2.80 | 0.80 | 4.80 | **0.006** |
| ≥ 150 min | 6.57 | 2.26 | 10.87 | **0.003** |  | 3.54 | 0.85 | 6.24 | **0.010** |  | 3.02 | 0.85 | 5.20 | **0.006** |
| Study (ref. SPEEDY) | | | | | | | | | | | | | | |
| ALSPAC | 12.98 | 10.55 | 15.40 | **<0.001** |  | 5.98 | 4.46 | 7.50 | **<0.001** |  | 7.00 | 5.77 | 8.22 | **<0.001** |
| CLAN | 21.15 | 17.25 | 25.05 | **<0.001** |  | 7.59 | 5.15 | 10.03 | **<0.001** |  | 13.56 | 11.59 | 15.53 | **<0.001** |
| Age | -2.99 | -3.84 | -2.14 | **<0.001** |  | -1.80 | -2.33 | -1.27 | **<0.001** |  | -1.19 | -1.62 | -0.76 | **<0.001** |
| Sex (ref. Male) | | | | | | | | | | | | | | |
| Female | -19.63 | -21.09 | -18.17 | **<0.001** |  | -13.08 | -14.00 | -12.17 | **<0.001** |  | -6.55 | -7.29 | -5.81 | **<0.001** |
| Maternal education (ref. High school) | | | | | | | | | | | | | | |
| College | -1.82 | -3.55 | -0.09 | **0.039** |  | -0.89 | -1.98 | 0.19 | 0.107 |  | -0.93 | -1.80 | -0.05 | **0.038** |
| University | -2.57 | -4.59 | -0.55 | **0.012** |  | -1.73 | -3.00 | -0.47 | **0.007** |  | -0.84 | -1.86 | 0.18 | 0.106 |
| Season (ref. Winter) | | | | | | | | | | | | | | |
| Spring | 6.93 | 4.67 | 9.19 | **<0.001** |  | 4.43 | 3.01 | 5.84 | **<0.001** |  | 2.51 | 1.36 | 3.65 | **<0.001** |
| Summer | 5.58 | 3.03 | 8.12 | **<0.001** |  | 3.86 | 2.27 | 5.45 | **<0.001** |  | 1.71 | 0.43 | 3.00 | **0.009** |
| Autumn | -0.89 | -3.65 | 1.87 | 0.529 |  | -0.67 | -2.40 | 1.06 | 0.447 |  | -0.22 | -1.61 | 1.18 | 0.762 |
| Monitor wear time (min/day) | 0.06 | 0.05 | 0.07 | **<0.001** |  | 0.04 | 0.03 | 0.05 | **<0.001** |  | 0.02 | 0.02 | 0.03 | **<0.001** |
| Constant | 36.39 | 23.63 | 49.15 | **<0.001** |  | 29.25 | 21.26 | 37.23 | **<0.001** |  | 7.14 | 0.69 | 13.58 | **0.030** |
| The models were adjusted for age, sex, maternal education, season, monitor wear time, and study.  ^†^**Bold**: Significance level at 5%.  ALSPAC = Avon Longitudinal Study of Parents and Children, CI = confidence interval, CLAN = Children Living in Active Neighbourhoods, MPA = moderate physical activity, MVPA = moderate-to-vigorous physical activity, SPEEDY = Sport, Physical activity and Eating behaviour: Environmental Determinants in Young people, VPA = vigorous physical activity. | | | | | | | | | | | | | | |
