# Supplementary material for: Cross-sectional and longitudinal associations of active travel, organised sport and physical education with accelerometer-assessed moderate-to-vigorous physical activity in young people: the International Children’s Accelerometry Database
Source: Int J Behav Nutr Phys Act. 2022 Apr 2;19:41. doi: 10.1186/s12966-022-01282-4 (PMC8977036; doi:10.1186/s12966-022-01282-4)
Supplement: Supplementary file 8 — Additional file 8. [file 12966_2022_1282_MOESM8_ESM.docx]

# Additional File 8

## Longitudinal association of domain-specific physical activity and covariates with daily accelerometer-assessed MVPA, MPA and VPA (N = 2302)

|  | **Change in MVPA** | | | |  | **Change in MPA** | | | |  | **Change in VPA** | | | |
| --- | --- | --- | --- | --- | --- | --- | --- | --- | --- | --- | --- | --- | --- | --- |
|  | *Coefficient* | *95% CI* | | *P-value^†^* |  | *Coefficient* | *95% CI* | | *P-value^†^* |  | *Coefficient* | *95% CI* | | *P-value^†^* |
|  |  | *Lower* | *Upper* |  |  |  | *Lower* | *Upper* |  |  |  | *Lower* | *Upper* |  |
| **Active travel** (ref. Other mode) | | | | | | | | | | | | | | |
| Active mode | 1.37 | -0.28 | 3.02 | 0.105 |  | 0.84 | -0.21 | 1.88 | 0.117 |  | 0.70 | -0.23 | 1.62 | 0.140 |
| Study (ref. SPEEDY) | | | | | | | | | | | | | | |
| ALSPAC | 5.05 | 2.61 | 7.50 | **<0.001** |  | 1.51 | -0.04 | 3.06 | 0.057 |  | 3.75 | 2.42 | 5.08 | **<0.001** |
| CLAN | -0.37 | -3.64 | 2.89 | 0.823 |  | 0.63 | -1.26 | 2.52 | 0.515 |  | -0.57 | -2.57 | 1.44 | 0.580 |
| Age | -0.42 | -1.18 | 0.33 | 0.270 |  | -1.00 | -1.44 | -0.55 | **<0.001** |  | 0.51 | 0.05 | 0.97 | **0.029** |
| Sex (ref. Male) | | | | | | | | | | | | | | |
| Female | -8.48 | -10.23 | -6.74 | **<0.001** |  | -4.36 | -5.45 | -3.28 | **<0.001** |  | -4.50 | -5.46 | -3.54 | **<0.001** |
| Maternal education (ref. High school) | | | | | | | | | | | | | | |
| College | -0.94 | -2.92 | 1.04 | 0.353 |  | -0.91 | -2.17 | 0.34 | 0.153 |  | -0.05 | -1.17 | 1.08 | 0.936 |
| University | -0.81 | -2.98 | 1.36 | 0.463 |  | -1.23 | -2.60 | 0.14 | 0.079 |  | 0.41 | -0.83 | 1.66 | 0.513 |
| Change in season (ref. Same) | | | | | | | | | | | | | | |
| Shorter & colder | -3.17 | -5.33 | -1.02 | **0.004** |  | -3.07 | -4.41 | -1.73 | **<0.001** |  | -0.04 | -1.24 | 1.15 | 0.942 |
| Longer & warmer | 1.57 | -0.79 | 3.93 | 0.191 |  | 0.77 | -0.70 | 2.24 | 0.303 |  | 0.71 | -0.61 | 2.03 | 0.292 |
| Change in monitor wear time (min/day) | 0.05 | 0.04 | 0.07 | **<0.001** |  | 0.03 | 0.03 | 0.04 | **<0.001** |  | 0.02 | 0.01 | 0.03 | **<0.001** |
| Follow-up duration (year) | -2.95 | -3.68 | -2.23 | **<0.001** |  | -2.30 | -2.78 | -1.81 | **<0.001** |  | -0.65 | -1.03 | -0.28 | **0.001** |
| Baseline MVPA/MPA/VPA* (min/day) | -0.56 | -0.61 | -0.52 | **<0.001** |  | -0.58 | -0.63 | -0.54 | **<0.001** |  | -0.59 | -0.64 | -0.54 | **<0.001** |
| Constant | 37.73 | 28.94 | 46.52 | **<0.001** |  | 35.52 | 30.06 | 40.98 | **<0.001** |  | 3.97 | -1.01 | 8.95 | 0.118 |
| **Organised sport** (ref. Never) | | | | | | | | | | | | | | |
| Occasionally | 0.25 | -2.68 | 3.19 | 0.866 |  | -0.21 | -2.13 | 1.72 | 0.832 |  | 0.51 | -1.12 | 2.14 | 0.541 |
| Sometimes | 0.28 | -2.66 | 3.23 | 0.850 |  | 0.54 | -1.42 | 2.49 | 0.589 |  | -0.16 | -1.81 | 1.48 | 0.845 |
| Often | 3.01 | -0.12 | 6.14 | 0.060 |  | 2.02 | 0.02 | 4.03 | **0.048** |  | 1.17 | -0.56 | 2.91 | 0.185 |
| Usually | 1.41 | -1.93 | 4.74 | 0.408 |  | 1.86 | -0.29 | 4.02 | 0.091 |  | -0.20 | -2.02 | 1.62 | 0.831 |
| Study (ref. SPEEDY) | | | | | | | | | | | | | | |
| ALSPAC | 5.10 | 2.65 | 7.55 | **<0.001** |  | 1.68 | 0.12 | 3.23 | **0.035** |  | 3.64 | 2.32 | 4.96 | **<0.001** |
| CLAN | -0.58 | -3.80 | 2.64 | 0.723 |  | 0.60 | -1.26 | 2.46 | 0.528 |  | -0.81 | -2.79 | 1.16 | 0.419 |
| Age | -0.47 | -1.23 | 0.29 | 0.223 |  | -1.05 | -1.49 | -0.61 | **<0.001** |  | 0.51 | 0.05 | 0.98 | **0.029** |
| Sex (ref. Male) | | | | | | | | | | | | | | |
| Female | -8.41 | -10.16 | -6.67 | **<0.001** |  | -4.34 | -5.41 | -3.26 | **<0.001** |  | -4.47 | -5.43 | -3.50 | **<0.001** |
| Maternal education (ref. High school) | | | | | | | | | | | | | | |
| College | -0.94 | -2.92 | 1.04 | 0.352 |  | -0.94 | -2.19 | 0.31 | 0.140 |  | -0.03 | -1.15 | 1.10 | 0.965 |
| University | -0.92 | -3.09 | 1.25 | 0.405 |  | -1.35 | -2.71 | 0.01 | 0.052 |  | 0.40 | -0.84 | 1.63 | 0.527 |
| Change in season (ref. Same) | | | | | | | | | | | | | | |
| Shorter & colder | -3.18 | -5.33 | -1.04 | **0.004** |  | -3.08 | -4.42 | -1.74 | **<0.001** |  | -0.05 | -1.25 | 1.15 | 0.934 |
| Longer & warmer | 1.53 | -0.82 | 3.88 | 0.201 |  | 0.75 | -0.71 | 2.21 | 0.315 |  | 0.69 | -0.63 | 2.01 | 0.306 |
| Change in monitor wear time (min/day) | 0.05 | 0.04 | 0.07 | **<0.001** |  | 0.03 | 0.03 | 0.04 | **<0.001** |  | 0.02 | 0.01 | 0.03 | **<0.001** |
| Follow-up duration (year) | -2.96 | -3.68 | -2.23 | **<0.001** |  | -2.31 | -2.79 | -1.82 | **<0.001** |  | -0.65 | -1.02 | -0.28 | **0.001** |
| Baseline MVPA/MPA/VPA* (min/day) | -0.56 | -0.61 | -0.52 | **<0.001** |  | -0.59 | -0.63 | -0.54 | **<0.001** |  | -0.58 | -0.63 | -0.53 | **<0.001** |
| Constant | 37.88 | 28.78 | 46.97 | **<0.001** |  | 35.85 | 30.14 | 41.56 | **<0.001** |  | 3.90 | -1.27 | 9.08 | 0.139 |
| **Physical education** (ref. 0-59 min) | | | | | | | | | | | | | | |
| 60-89 min | 0.50 | -3.84 | 4.83 | 0.823 |  | 0.35 | -2.29 | 3.00 | 0.793 |  | 0.24 | -2.13 | 2.62 | 0.841 |
| 90-119 min | -0.05 | -4.41 | 4.31 | 0.983 |  | 0.44 | -2.26 | 3.14 | 0.750 |  | -0.36 | -2.69 | 1.97 | 0.763 |
| 120-149 min | 0.69 | -3.64 | 5.03 | 0.754 |  | 0.79 | -1.89 | 3.46 | 0.564 |  | 0.03 | -2.30 | 2.37 | 0.977 |
| ≥ 150 min | -0.08 | -4.73 | 4.56 | 0.972 |  | 0.27 | -2.65 | 3.19 | 0.857 |  | -0.25 | -2.74 | 2.24 | 0.842 |
| Study (ref. SPEEDY) | | | | | | | | | | | | | | |
| ALSPAC | 5.11 | 2.45 | 7.77 | **<0.001** |  | 1.65 | -0.04 | 3.34 | 0.055 |  | 3.67 | 2.22 | 5.13 | **<0.001** |
| CLAN | -0.48 | -4.43 | 3.48 | 0.813 |  | 0.85 | -1.50 | 3.20 | 0.477 |  | -0.85 | -3.16 | 1.46 | 0.471 |
| Age | -0.41 | -1.19 | 0.38 | 0.311 |  | -1.02 | -1.48 | -0.55 | **<0.001** |  | 0.54 | 0.07 | 1.01 | **0.024** |
| Sex (ref. Male) | | | | | | | | | | | | | | |
| Female | -8.46 | -10.21 | -6.72 | **<0.001** |  | -4.35 | -5.43 | -3.26 | **<0.001** |  | -4.51 | -5.47 | -3.55 | **<0.001** |
| Maternal education (ref. High school) | | | | | | | | | | | | | | |
| College | -0.93 | -2.92 | 1.06 | 0.358 |  | -0.92 | -2.17 | 0.34 | 0.152 |  | -0.04 | -1.17 | 1.10 | 0.951 |
| University | -0.89 | -3.07 | 1.29 | 0.423 |  | -1.28 | -2.65 | 0.09 | 0.066 |  | 0.37 | -0.88 | 1.63 | 0.560 |
| Change in season (ref. Same) | | | | | | | | | | | | | | |
| Shorter & colder | -3.17 | -5.33 | -1.02 | **0.004** |  | -3.08 | -4.42 | -1.73 | **<0.001** |  | -0.04 | -1.23 | 1.16 | 0.953 |
| Longer & warmer | 1.60 | -0.75 | 3.96 | 0.181 |  | 0.80 | -0.67 | 2.26 | 0.287 |  | 0.72 | -0.60 | 2.04 | 0.286 |
| Change in monitor wear time (min/day) | 0.05 | 0.04 | 0.07 | **<0.001** |  | 0.03 | 0.03 | 0.04 | **<0.001** |  | 0.02 | 0.01 | 0.03 | **<0.001** |
| Follow-up duration (year) | -2.94 | -3.67 | -2.22 | **<0.001** |  | -2.29 | -2.78 | -1.81 | **<0.001** |  | -0.65 | -1.02 | -0.27 | **0.001** |
| Baseline MVPA/MPA/VPA* (min/day) | -0.56 | -0.60 | -0.52 | **<0.001** |  | -0.58 | -0.62 | -0.53 | **<0.001** |  | -0.58 | -0.63 | -0.53 | **<0.001** |
| Constant | 37.46 | 28.33 | 46.59 | **<0.001** |  | 35.29 | 29.64 | 40.94 | **<0.001** |  | 4.02 | -1.27 | 9.30 | 0.136 |
| The models were adjusted for age, sex, maternal education, change in season, change in monitor wear time, follow-up duration, baseline MVPA/MPA/VPA and study.  ^†^**Bold**: Significance level at 5%.  *The same intensity as the outcome measure (MVPA/MPA/VPA) was only included in the model.  ALSPAC = Avon Longitudinal Study of Parents and Children, CI = confidence interval, CLAN = Children Living in Active Neighbourhoods, MPA = moderate physical activity, MVPA = moderate-to-vigorous physical activity, SPEEDY = Sport, Physical activity and Eating behaviour: Environmental Determinants in Young people, VPA = vigorous physical activity. | | | | | | | | | | | | | | |
