# Supplementary material for: Cross-sectional and longitudinal associations of active travel, organised sport and physical education with accelerometer-assessed moderate-to-vigorous physical activity in young people: the International Children’s Accelerometry Database
Source: Int J Behav Nutr Phys Act. 2022 Apr 2;19:41. doi: 10.1186/s12966-022-01282-4 (PMC8977036; doi:10.1186/s12966-022-01282-4)
Supplement: Supplementary file 9 — Additional file 9. [file 12966_2022_1282_MOESM9_ESM.docx]

# Additional File 9

## Cross-sectional association of standardised domain-specific physical activity and covariates with daily accelerometer-assessed MVPA, MPA and VPA (N = 3871)

|  | **MVPA** | | | |  | | **MPA** | | | | |  | | **VPA** | | | | |  |
| --- | --- | --- | --- | --- | --- | --- | --- | --- | --- | --- | --- | --- | --- | --- | --- | --- | --- | --- | --- |
|  | *Beta coefficient* | *95% CI* | | *P-value^†^* | |  | | *Beta coefficient* | *95% CI* | | *P-value^†^* | |  | | *Beta coefficient* | *95% CI* | | *P-value^†^* | |
|  |  | *Lower* | *Upper* |  |  |  | |  | *Lower* | *Upper* |  |  |  | |  | *Lower* | *Upper* |  |  |
| **Active travel** | 3.46 | 2.73 | 4.19 | **<0.001** | |  | | 2.29 | 1.83 | 2.75 | **<0.001** | |  | | 1.17 | 0.80 | 1.54 | **<0.001** | |
| Study (ref. SPEEDY) | | | | | | | | | | | | | | | | | | |  |
| ALSPAC | 12.48 | 10.30 | 14.67 | **<0.001** | |  | | 5.97 | 4.60 | 7.33 | **<0.001** | |  | | 6.51 | 5.40 | 7.62 | **<0.001** | |
| CLAN | 20.04 | 16.80 | 23.27 | **<0.001** | |  | | 7.51 | 5.49 | 9.53 | **<0.001** | |  | | 12.53 | 10.88 | 14.17 | **<0.001** | |
| Age | -2.64 | -3.45 | -1.82 | **<0.001** | |  | | -1.60 | -2.11 | -1.08 | **<0.001** | |  | | -1.04 | -1.46 | -0.63 | **<0.001** | |
| Sex (ref. Male) | | | | | | | | | | | | | | | | | | |  |
| Female | -19.39 | -20.83 | -17.94 | **<0.001** | |  | | -12.94 | -13.84 | -12.03 | **<0.001** | |  | | -6.45 | -7.18 | -5.71 | **<0.001** | |
| Maternal education (ref. High school) | | | | | | | | | | | | | | | | | | |  |
| College | -1.58 | -3.30 | 0.13 | 0.071 | |  | | -0.74 | -1.81 | 0.34 | 0.178 | |  | | -0.85 | -1.72 | 0.03 | 0.057 | |
| University | -1.62 | -3.62 | 0.37 | 0.111 | |  | | -1.14 | -2.38 | 0.11 | 0.075 | |  | | -0.49 | -1.50 | 0.53 | 0.347 | |
| Season (ref. Winter) | | | | | | | | | | | | | | | | | | |  |
| Spring | 6.79 | 4.55 | 9.02 | **<0.001** | |  | | 4.37 | 2.98 | 5.77 | **<0.001** | |  | | 2.42 | 1.28 | 3.55 | **<0.001** | |
| Summer | 5.29 | 2.77 | 7.80 | **<0.001** | |  | | 3.68 | 2.11 | 5.25 | **<0.001** | |  | | 1.60 | 0.33 | 2.88 | **0.014** | |
| Autumn | -1.02 | -3.75 | 1.71 | 0.465 | |  | | -0.75 | -2.46 | 0.96 | 0.389 | |  | | -0.27 | -1.66 | 1.12 | 0.704 | |
| Monitor wear time (min/day) | 0.06 | 0.05 | 0.07 | **<0.001** | |  | | 0.04 | 0.03 | 0.04 | **<0.001** | |  | | 0.02 | 0.02 | 0.03 | **<0.001** | |
| Constant | 37.81 | 25.47 | 50.15 | **<0.001** | |  | | 29.38 | 21.66 | 37.09 | **<0.001** | |  | | 8.43 | 2.16 | 14.70 | **0.021** | |
| **Organised sport** | 3.81 | 3.06 | 4.56 | **<0.001** | |  | | 2.35 | 1.88 | 2.82 | **<0.001** | |  | | 1.46 | 1.08 | 1.84 | **<0.001** | |
| Study (ref. SPEEDY) | | | | | | | | | | | | | | | | | | |  |
| ALSPAC | 13.79 | 11.60 | 15.99 | **<0.001** | |  | | 6.78 | 5.40 | 8.15 | **<0.001** | |  | | 7.02 | 5.90 | 8.13 | **<0.001** | |
| CLAN | 19.33 | 16.11 | 22.55 | **<0.001** | |  | | 7.02 | 5.00 | 9.03 | **<0.001** | |  | | 12.31 | 10.68 | 13.94 | **<0.001** | |
| Age | -2.96 | -3.78 | -2.15 | **<0.001** | |  | | -1.80 | -2.31 | -1.29 | **<0.001** | |  | | -1.16 | -1.58 | -0.75 | **<0.001** | |
| Sex (ref. Male) | | | | | | | | | | | | | | | | | | |  |
| Female | -18.86 | -20.31 | -17.41 | **<0.001** | |  | | -12.63 | -13.54 | -11.72 | **<0.001** | |  | | -6.24 | -6.97 | -5.50 | **<0.001** | |
| Maternal education (ref. High school) | | | | | | | | | | | | | | | | | | |  |
| College | -2.07 | -3.78 | -0.35 | **0.018** | |  | | -1.04 | -2.12 | 0.03 | 0.056 | |  | | -1.02 | -1.89 | -0.15 | **0.021** | |
| University | -2.84 | -4.83 | -0.85 | **0.005** | |  | | -1.92 | -3.16 | -0.67 | **0.003** | |  | | -0.92 | -1.93 | 0.09 | 0.074 | |
| Season (ref. Winter) | | | | | | | | | | | | | | | | | | |  |
| Spring | 6.60 | 4.37 | 8.83 | **<0.001** | |  | | 4.26 | 2.86 | 5.66 | **<0.001** | |  | | 2.34 | 1.21 | 3.48 | **<0.001** | |
| Summer | 5.20 | 2.69 | 7.71 | **<0.001** | |  | | 3.64 | 2.06 | 5.21 | **<0.001** | |  | | 1.56 | 0.29 | 2.84 | **0.016** | |
| Autumn | -1.15 | -3.88 | 1.57 | 0.407 | |  | | -0.83 | -2.54 | 0.88 | 0.340 | |  | | -0.32 | -1.71 | 1.06 | 0.647 | |
| Monitor wear time (min/day) | 0.06 | 0.05 | 0.07 | **<0.001** | |  | | 0.04 | 0.03 | 0.05 | **<0.001** | |  | | 0.02 | 0.02 | 0.03 | **<0.001** | |
| Constant | 39.76 | 27.44 | 52.08 | **<0.001** | |  | | 30.64 | 22.93 | 38.35 | **<0.001** | |  | | 9.12 | 2.87 | 15.37 | **0.004** | |
| **Physical education** | 0.82 | -0.02 | 1.66 | 0.056 | |  | | 0.34 | -0.18 | 0.87 | 0.199 | |  | | 0.47 | 0.05 | 0.90 | **0.029** | |
| Study (ref. SPEEDY) | | | | | | | | | | | | | | | | | | |  |
| ALSPAC | 13.33 | 10.94 | 15.73 | **<0.001** | |  | | 6.32 | 4.82 | 7.81 | **<0.001** | |  | | 7.02 | 5.81 | 8.22 | **<0.001** | |
| CLAN | 20.52 | 16.82 | 24.22 | **<0.001** | |  | | 7.42 | 5.10 | 9.74 | **<0.001** | |  | | 13.10 | 11.23 | 14.97 | **<0.001** | |
| Age | -2.89 | -3.74 | -2.05 | **<0.001** | |  | | -1.73 | -2.26 | -1.20 | **<0.001** | |  | | -1.17 | -1.59 | -0.74 | **<0.001** | |
| Sex (ref. Male) | | | | | | | | | | | | | | | | | | |  |
| Female | -19.66 | -21.12 | -18.19 | **<0.001** | |  | | -13.11 | -14.02 | -12.19 | **<0.001** | |  | | -6.55 | -7.29 | -5.81 | **<0.001** | |
| Maternal education (ref. High school) | | | | | | | | | | | | | | | | | | |  |
| College | -1.83 | -3.56 | -0.09 | **0.039** | |  | | -0.89 | -1.97 | 0.20 | 0.109 | |  | | -0.94 | -1.82 | -0.06 | **0.035** | |
| University | -2.52 | -4.54 | -0.51 | **0.014** | |  | | -1.69 | -2.96 | -0.43 | **0.009** | |  | | -0.83 | -1.85 | 0.19 | 0.110 | |
| Season (ref. Winter) | | | | | | | | | | | | | | | | | | |  |
| Spring | 6.87 | 4.61 | 9.13 | **<0.001** | |  | | 4.42 | 3.00 | 5.83 | **<0.001** | |  | | 2.45 | 1.31 | 3.59 | **<0.001** | |
| Summer | 5.45 | 2.91 | 7.99 | **<0.001** | |  | | 3.78 | 2.19 | 5.37 | **<0.001** | |  | | 1.67 | 0.39 | 2.95 | **0.011** | |
| Autumn | -0.92 | -3.68 | 1.84 | 0.514 | |  | | -0.69 | -2.42 | 1.03 | 0.431 | |  | | -0.23 | -1.62 | 1.17 | 0.751 | |
| Monitor wear time (min/day) | 0.06 | 0.05 | 0.07 | **<0.001** | |  | | 0.04 | 0.03 | 0.04 | **<0.001** | |  | | 0.02 | 0.02 | 0.03 | **<0.001** | |
| Constant | 40.23 | 27.71 | 52.75 | **<0.001** | |  | | 30.72 | 22.88 | 38.55 | **<0.001** | |  | | 9.52 | 3.20 | 15.84 | **0.003** | |
| The models were adjusted for age, sex, maternal education, season, monitor wear time, and study.  ^†^**Bold**: Significance level at 5%.  ALSPAC = Avon Longitudinal Study of Parents and Children, CI = confidence interval, CLAN = Children Living in Active Neighbourhoods, MPA = moderate physical activity, MVPA = moderate-to-vigorous physical activity, SPEEDY = Sport, Physical activity and Eating behaviour: Environmental Determinants in Young people, VPA = vigorous physical activity. | | | | | | | | | | | | | | | | | | |  |
