# Supplementary material for: Cross-sectional and longitudinal associations of active travel, organised sport and physical education with accelerometer-assessed moderate-to-vigorous physical activity in young people: the International Children’s Accelerometry Database
Source: Int J Behav Nutr Phys Act. 2022 Apr 2;19:41. doi: 10.1186/s12966-022-01282-4 (PMC8977036; doi:10.1186/s12966-022-01282-4)
Supplement: Supplementary file 10 — Additional file 10. [file 12966_2022_1282_MOESM10_ESM.docx]

# Additional File 10

## Longitudinal association of standardised domain-specific physical activity and covariates with daily accelerometer-assessed MVPA, MPA and VPA (N = 2302)

|  | **Change in MVPA** | | | |  | **Change in MPA** | | | |  | **Change in VPA** | | | |
| --- | --- | --- | --- | --- | --- | --- | --- | --- | --- | --- | --- | --- | --- | --- |
|  | *Beta coefficient* | *95% CI* | | *P-value^†^* |  | *Beta coefficient* | *95% CI* | | *P-value^†^* |  | *Beta coefficient* | *95% CI* | | *P-value^†^* |
|  |  | *Lower* | *Upper* |  |  |  | *Lower* | *Upper* |  |  |  | *Lower* | *Upper* |  |
| **Active travel** | 0.68 | -0.14 | 1.50 | 0.105 |  | 0.41 | -0.10 | 0.93 | 0.117 |  | 0.35 | -0.11 | 0.80 | 0.140 |
| Study (ref. SPEEDY) | | | | | | | | | | | | | | |
| ALSPAC | 5.05 | 2.61 | 7.50 | **<0.001** |  | 1.51 | -0.04 | 3.06 | 0.057 |  | 3.75 | 2.42 | 5.08 | **<0.001** |
| CLAN | -0.37 | -3.64 | 2.89 | 0.823 |  | 0.63 | -1.26 | 2.52 | 0.515 |  | -0.57 | -2.57 | 1.44 | 0.580 |
| Age | -0.42 | -1.18 | 0.33 | 0.270 |  | -1.00 | -1.44 | -0.55 | **<0.001** |  | 0.51 | 0.05 | 0.97 | **0.029** |
| Sex (ref. Male) | | | | | | | | | | | | | | |
| Female | -8.48 | -10.23 | -6.74 | **<0.001** |  | -4.36 | -5.45 | -3.28 | **<0.001** |  | -4.50 | -5.46 | -3.54 | **<0.001** |
| Maternal education (ref. High school) | | | | | | | | | | | | | | |
| College | -0.94 | -2.92 | 1.04 | 0.353 |  | -0.91 | -2.17 | 0.34 | 0.153 |  | -0.05 | -1.17 | 1.08 | 0.936 |
| University | -0.81 | -2.98 | 1.36 | 0.463 |  | -1.23 | -2.60 | 0.14 | 0.079 |  | 0.41 | -0.83 | 1.66 | 0.513 |
| Change in season (ref. Same) | | | | | | | | | | | | | | |
| Shorter & colder | -3.17 | -5.33 | -1.02 | **0.004** |  | -3.07 | -4.41 | -1.73 | **<0.001** |  | -0.04 | -1.24 | 1.15 | 0.942 |
| Longer & warmer | 1.57 | -0.79 | 3.93 | 0.191 |  | 0.77 | -0.70 | 2.24 | 0.303 |  | 0.71 | -0.61 | 2.03 | 0.292 |
| Change in monitor wear time (min/day) | 0.05 | 0.04 | 0.07 | **<0.001** |  | 0.03 | 0.03 | 0.04 | **<0.001** |  | 0.02 | 0.01 | 0.03 | **<0.001** |
| Follow-up duration (year) | -2.95 | -3.68 | -2.23 | **<0.001** |  | -2.30 | -2.78 | -1.81 | **<0.001** |  | -0.65 | -1.03 | -0.28 | **0.001** |
| Baseline MVPA/MPA/VPA* (min/day) | -0.56 | -0.61 | -0.52 | **<0.001** |  | -0.58 | -0.63 | -0.54 | **<0.001** |  | -0.59 | -0.64 | -0.54 | **<0.001** |
| Constant | 38.33 | 29.56 | 47.11 | **<0.001** |  | 35.88 | 30.44 | 41.33 | **<0.001** |  | 4.28 | -0.70 | 9.25 | 0.092 |
| **Organised sport** | 0.85 | -0.03 | 1.72 | 0.059 |  | 0.90 | 0.35 | 1.45 | **0.001** |  | 0.03 | -0.45 | 0.52 | 0.891 |
| Study (ref. SPEEDY) | | | | | | | | | | | | | | |
| ALSPAC | 5.23 | 2.78 | 7.68 | **<0.001** |  | 1.73 | 0.18 | 3.29 | **0.029** |  | 3.71 | 2.39 | 5.03 | 0.000 |
| CLAN | -0.58 | -3.80 | 2.64 | 0.724 |  | 0.55 | -1.31 | 2.42 | 0.559 |  | -0.76 | -2.73 | 1.21 | 0.451 |
| Age | -0.47 | -1.22 | 0.29 | 0.228 |  | -1.04 | -1.48 | -0.60 | **<0.001** |  | 0.51 | 0.05 | 0.97 | **0.031** |
| Sex (ref. Male) | | | | | | | | | | | | | | |
| Female | -8.45 | -10.20 | -6.71 | **<0.001** |  | -4.35 | -5.43 | -3.27 | **<0.001** |  | -4.50 | -5.46 | -3.54 | **<0.001** |
| Maternal education (ref. High school) | | | | | | | | | | | | | | |
| College | -1.03 | -3.01 | 0.95 | 0.306 |  | -1.00 | -2.25 | 0.25 | 0.117 |  | -0.06 | -1.19 | 1.07 | 0.917 |
| University | -1.06 | -3.23 | 1.10 | 0.335 |  | -1.45 | -2.81 | -0.09 | **0.037** |  | 0.35 | -0.89 | 1.60 | 0.578 |
| Change in season (ref. Same) | | | | | | | | | | | | | | |
| Shorter & colder | -3.20 | -5.35 | -1.04 | **0.004** |  | -3.09 | -4.43 | -1.75 | **<0.001** |  | -0.05 | -1.25 | 1.14 | 0.931 |
| Longer & warmer | 1.61 | -0.74 | 3.95 | 0.179 |  | 0.79 | -0.67 | 2.25 | 0.288 |  | 0.72 | -0.60 | 2.04 | 0.284 |
| Change in monitor wear time (min/day) | 0.05 | 0.04 | 0.07 | **<0.001** |  | 0.03 | 0.03 | 0.04 | **<0.001** |  | 0.02 | 0.01 | 0.03 | **<0.001** |
| Follow-up duration (year) | -2.96 | -3.69 | -2.24 | **<0.001** |  | -2.31 | -2.80 | -1.82 | **<0.001** |  | -0.65 | -1.02 | -0.28 | **0.001** |
| Baseline MVPA/MPA/VPA* (min/day) | -0.56 | -0.61 | -0.52 | **<0.001** |  | -0.59 | -0.63 | -0.54 | **<0.001** |  | -0.58 | -0.64 | -0.53 | **<0.001** |
| Constant | 38.89 | 30.05 | 47.74 | **<0.001** |  | 36.61 | 31.16 | 42.05 | **<0.001** |  | 4.31 | -0.69 | 9.31 | 0.091 |
| **Physical education** | 0.02 | -0.87 | 0.91 | 0.962 |  | 0.10 | -0.47 | 0.66 | 0.738 |  | -0.06 | -0.56 | 0.43 | 0.803 |
| Study (ref. SPEEDY) | | | | | | | | | | | | | | |
| ALSPAC | 4.97 | 2.34 | 7.59 | **<0.001** |  | 1.55 | -0.12 | 3.23 | 0.069 |  | 3.63 | 2.21 | 5.05 | **<0.001** |
| CLAN | -0.72 | -4.44 | 2.99 | 0.703 |  | 0.61 | -1.61 | 2.83 | 0.589 |  | -0.90 | -3.10 | 1.30 | 0.424 |
| Age | -0.42 | -1.20 | 0.35 | 0.284 |  | -1.01 | -1.47 | -0.55 | **<0.001** |  | 0.52 | 0.06 | 0.99 | **0.028** |
| Sex (ref. Male) | | | | | | | | | | | | | | |
| Female | -8.44 | -10.18 | -6.69 | **<0.001** |  | -4.34 | -5.42 | -3.25 | **<0.001** |  | -4.49 | -5.45 | -3.54 | **<0.001** |
| Maternal education (ref. High school) | | | | | | | | | | | | | | |
| College | -0.96 | -2.95 | 1.02 | 0.341 |  | -0.93 | -2.19 | 0.32 | 0.145 |  | -0.05 | -1.18 | 1.08 | 0.927 |
| University | -0.92 | -3.10 | 1.25 | 0.405 |  | -1.32 | -2.69 | 0.05 | 0.060 |  | 0.37 | -0.88 | 1.62 | 0.562 |
| Change in season (ref. Same) | | | | | | | | | | | | | | |
| Shorter & colder | -3.20 | -5.35 | -1.04 | **0.004** |  | -3.09 | -4.43 | -1.74 | **<0.001** |  | -0.05 | -1.25 | 1.15 | 0.932 |
| Longer & warmer | 1.61 | -0.74 | 3.95 | 0.180 |  | 0.80 | -0.67 | 2.26 | 0.287 |  | 0.72 | -0.60 | 2.04 | 0.284 |
| Change in monitor wear time (min/day) | 0.05 | 0.04 | 0.07 | **<0.001** |  | 0.03 | 0.03 | 0.04 | **<0.001** |  | 0.02 | 0.01 | 0.03 | **<0.001** |
| Follow-up duration (year) | -2.95 | -3.67 | -2.22 | **<0.001** |  | -2.29 | -2.78 | -1.80 | **<0.001** |  | -0.65 | -1.02 | -0.28 | **0.001** |
| Baseline MVPA/MPA/VPA* (min/day) | -0.56 | -0.60 | -0.52 | **<0.001** |  | -0.58 | -0.62 | -0.53 | **<0.001** |  | -0.58 | -0.63 | -0.53 | **<0.001** |
| Constant | 38.14 | 29.22 | 47.07 | **<0.001** |  | 35.87 | 30.31 | 41.43 | **<0.001** |  | 4.19 | -0.83 | 9.21 | 0.102 |
| The models were adjusted for age, sex, maternal education, change in season, change in monitor wear time, follow-up duration, baseline MVPA/MPA/VPA and study.  ^†^**Bold**: Significance level at 5%.  *The same intensity as the outcome measure (MVPA/MPA/VPA) was only included in the model.  ALSPAC = Avon Longitudinal Study of Parents and Children, CI = confidence interval, CLAN = Children Living in Active Neighbourhoods, MPA = moderate physical activity, MVPA = moderate-to-vigorous physical activity, SPEEDY = Sport, Physical activity and Eating behaviour: Environmental Determinants in Young people, VPA = vigorous physical activity. | | | | | | | | | | | | | | |
